# Disentangling Metaphor from Context: An ERP Study

**DOI:** 10.3389/fpsyg.2016.00559

**Published:** 2016-05-03

**Authors:** Valentina Bambini, Chiara Bertini, Walter Schaeken, Alessandra Stella, Francesco Di Russo

**Affiliations:** ^1^Center for Neurocognition, Epistemology and theoretical Syntax (NEtS), Institute for Advanced Study (IUSS)Pavia, Italy; ^2^Laboratorio di Linguistica “G. Nencioni”, Scuola Normale SuperiorePisa, Italy; ^3^Laboratory of Experimental Psychology, KU LeuvenLeuven, Belgium; ^4^Department of Movement, Human and Health Sciences, University of Rome “Foro Italico”Rome, Italy; ^5^Istituti di Ricovero e Cura a Carattere Scientifico Santa Lucia FoundationRome, Italy

**Keywords:** metaphor, context, pragmatics, neuropragmatics, experimental pragmatics, N400, P600

## Abstract

A large body of electrophysiological literature showed that metaphor comprehension elicits two different event-related brain potential responses, namely the so-called N400 and P600 components. Yet most of these studies test metaphor in isolation while in natural conversation metaphors do not come out of the blue but embedded in linguistic and extra-linguistic context. This study aimed at assessing the role of context in the metaphor comprehension process. We recorded EEG activity while participants were presented with metaphors and equivalent literal expressions in a minimal context (Experiment 1) and in a supportive context where the word expressing the ground between the metaphor's topic and vehicle was made explicit (Experiment 2). The N400 effect was visible only in minimal context, whereas the P600 was visible both in the absence and in the presence of contextual cues. These findings suggest that the N400 observed for metaphor is related to contextual aspects, possibly indexing contextual expectations on upcoming words that guide lexical access and retrieval, while the P600 seems to reflect truly pragmatic interpretative processes needed to make sense of a metaphor and derive the speaker's meaning, also in the presence of contextual cues. In sum, previous information in the linguistic context biases toward a metaphorical interpretation but does not suppress interpretative pragmatic mechanisms to establish the intended meaning.

## Introduction

While understanding language in the context of communication, comprehenders have to infer the so-called speaker's meaning, i.e., what the speaker intends to communicate, which is vastly underdetermined by the literal meaning of words and sentences. The speaker's intended meaning is the result of a pragmatic inference exploiting world knowledge, the context, and the lexical meaning of the expression. Metaphor offers a major example of the gap between the literal meaning and the speaker's meaning, and describing how this gap is bridged in the mind/brain of language users is one of the major concerns of experimental pragmatics and neuropragmatics (Bambini, 2010; Bambini and Bara, 2012; Hagoort and Levinson, 2014; Grossman and Noveck, 2015).

With respect to processing, metaphor has been studied mainly in relation to the steps of comprehension. Positions are traditionally divided into two main models according to whether the access to figurative meaning is considered indirect, i.e., passing through a first stage where the literal meaning is represented, or direct. The indirect access model is linked to the view of classic scholars in pragmatics, namely Grice and Searle, and supported by evidence of longer reaction times for metaphorical as compared to literal expressions (Janus and Bever, 1985). Conversely, the direct access view claims that, with appropriate context, people take no longer to understand metaphors than to understand comparable literal language (Gibbs, 1994). Somehow in between, the Graded Salience Hypothesis claims that the direct access is influenced by the salience degree of the stimuli (Giora, 2003).

When the event-related potential (ERP) electrophysiological technique started to be used to investigate how metaphor comprehension unfolds over time, the issue of the processing steps was revived in terms of ERP components (Bambini and Resta, 2012; Rataj, 2014). Two components have been commonly reported for metaphors, namely a centro-parietal negativity (N400) and a later parietal positivity (P600/LPC) (Pynte et al., 1996; Coulson and Van Petten, 2002; De Grauwe et al., 2010; Schmidt-Snoek et al., 2015). The functional roles of these components in language processing are diverse. The N400 is generally linked to meaning processing, in relation to a plethora of stimulus types (Kutas and Federmeier, 2011). The P600, originally linked to syntactic reanalysis, is nowadays assumed to reflect also semantic and interpretation processes, such as sentence-level interpretation conflicts (Frenzel et al., 2011) and integration in the wider discourse model and communicative context (Brouwer et al., 2012).

When considered with respect to metaphor, defining the functional significance of these ERP components becomes even more complex, and entrenched with the debate over the direct vs. indirect account. Most studies reported a biphasic N400-P600 effect, assumed to link different stages in conceptual mapping (Coulson and Van Petten, 2002; De Grauwe et al., 2010). Other authors reported an N400 response only (Pynte et al., 1996), or a P600 only, described as a form of a reanalysis stage (Yang et al., 2013). Globally, studies with a biphasic pattern or a later effect tend to favor the indirect view, while studies focusing on the N400 argue against the indirect model. In addition, one important result evidenced in the literature is that the ERP components elicited by metaphor are modulated by the degree of conventionality of the expression, also known as familiarity (Rataj, 2014). For instance, novel metaphors seem to elicit larger N400 amplitude than conventional metaphors (Arzouan et al., 2007a; Lai et al., 2009), which might suggest an indirect access for the formers and a direct access for the latters, in line with the Graded Salience Hypothesis. Moreover, it seems that conventionality affects the type of processes indexed in the N400 (Lai and Curran, 2013). This complex scenario casts doubts on the specificity of the effects reported for metaphor, by highlighting the need of carefully controlling for confounding variables such as familiarity, and definitely leaves the issue of direct/indirect access to metaphorical meaning unsolved.

It is indeed very likely that standard comprehension tasks like those employed in the studies above, while allowing to disentangle different phases of processing, cannot answer the question whether the literal meaning plays a role. A recent study employed masked priming during EEG to explore the issue for metaphor and metonymy (Weiland et al., 2014). This technique proved useful to tap into early phases of processing (Schumacher et al., 2012), and might shed light on the hypothesis of an early literal stage. Results showed that, when literal meaning of metaphorically used words is primed (e.g., priming *hyenas* with *furry* in the metaphor *Those lobbyists are hyenas*), the amplitude of the N400 is reduced with respect to the unprimed condition, thus facilitating rather than interfering with the comprehension process. This speaks in favor of the involvement of literal meaning aspects in the N400 phase, and supports the indirect view, or at least the idea of the lingering of the literal meaning in early phases, consistently with recent theoretical proposals (Carston, 2010b) and behavioral priming studies (Rubio Fernandez, 2007).

One important issue when speaking of metaphor and pragmatics is context. Context is constitutive in pragmatics, where it is assumed to influence the comprehension process by adjusting meanings and shaping inferences. In natural use of language, metaphors occur in the context of a conversation, exploiting background knowledge as well as the previous discourse shared by speakers to base the non-literal use. However, electrophysiological studies have rarely considered the issue of context with the aim of explicitly assessing its role. Pynte et al. (1996) varied the contextual support in the experimental stimuli, comparing familiar metaphors with supportive context and unfamiliar metaphors with non-supportive context, but this study does not help in disentangling the role of context in the comprehension of metaphorical meanings, as the manipulation mixed familiarity and context. Interesting hints into the role of context are provided in Yang et al. (2013). Through a word-to-sentence matching paradigm, the authors compared metaphorical and literal sentences with different probe words, which might work as contextual priming. The results evidenced a modulation of the P600, in the absence of N400 effect (Yang et al., 2013). Apart from this, stimuli employed in the literature are mostly limited to metaphors in the “A is B” sentence form or metaphorical word pairs with no supportive cues.

Other information comes from research on metonymy, where the manipulation of context was shown to directly influence the N400. The resolution of metonymic shift (e.g., *The ham sandwich wants to pay*) evoked a biphasic N400-LPC pattern when presented in minimal context (Schumacher, 2014), while only a LPC effect is visible when the linguistic context is supportive (e.g., already activating the restaurant semantic field) (Schumacher, 2011). Leaving aside the case of non-literal language, the issue of context is indeed the topic of a large body of investigation in the field, in particular with respect to the N400 components (van Berkum, 2009; Schumacher, 2012). The N400 seems to be sensitive to different types of context, including sentence level information (Hoeks et al., 2004; Federmeier et al., 2007) as well as larger discourse (Nieuwland and van Berkum, 2006), and non-linguistic information such as world knowledge (Hagoort et al., 2004) and speaker's identity (van Berkum et al., 2008). Recently, the literature hosted a debate between different views of the N400 (Lau et al., 2008; Hoeks and Brouwer, 2014). Some assume that the N400 reflects lexical access (Lau et al., 2009), other link the N400 to predictive mechanisms (Federmeier et al., 2007; Van Petten and Luka, 2012). In both cases context is crucial: in the lexical view context facilitates access and retrieval of stored information, in the prediction view context supports the ease of pre-activation and integration of meaning.

Also the P600/LPC has been described as context-sensitive. First, it is reported for several typically pragmatic phenomena that depend on context, such as irony (Regel et al., 2011; Spotorno et al., 2013), indirect request (Coulson and Lovett, 2010), jokes (Coulson and Kutas, 2001), as well as ambiguous idioms processing (Canal et al., 2015), question/answer pairs and other aspects of conversation and discourse (Hoeks et al., 2013; Hoeks and Brouwer, 2014). Second, context-based mechanisms such as expectation (Davenport and Coulson, 2011; Van Petten and Luka, 2012) and integration (Brouwer and Hoeks, 2013; Hoeks and Brouwer, 2014) have been advocated to describe the P600 as well.

Considering the literature on metaphor and the literature on context, questions arise whether context specifically affects the N400 observed for metaphor, and whether the P600 is also affected. The present study aims at exploring these issues by disentangling the benefit of linguistic context from the global process of understanding the speaker's meaning conveyed in a metaphor. To this purpose, we run two experiments where metaphors and corresponding literal sentences were presented in a minimal context (Experiment 1) and in a supportive context (Experiment 2). Supportive context was represented by the metaphor's ground, i.e., a word that expresses the relation between the metaphor's topic (the subject of the metaphor) and vehicle (the term used metaphorically). For instance, in the metaphor “Mary is a gem,” Mary is the topic, gem is the vehicle, and the ground is that Mary is precious or valued (End, 1986). This type of contextual information, which resembles natural occurrences of metaphors where the figurative use arises based on elements in the previous discourse or in the communicative situation, was already used in behavioral paradigms, producing a facilitation of the comprehension process (Gildea and Glucksberg, 1983). In order to avoid confounding effects due to familiarity, we employed non-lexicalized metaphors, and we checked for familiarity as a potentially confounding variable. Based on previous ERP studies on metaphor and on the literature on context effects, our prediction was twofold: (i) we expected to replicate the biphasic patterns observed in several studies for metaphors in minimal context; (ii) we expected context to reduce the N400 and possibly affect the P600. Results could also shed light on the functional characteristics of the components.

As a second aim of the study, we addressed the issue of localization. The source of the brain response to metaphor comprehension has been widely discussed in the imaging literature. While early studies highlighted the role of the right hemisphere (Bottini et al., 1994), later studies failed in reporting a right hemisphere advantage (Rapp et al., 2007) or evidenced a bilateral pattern (Bambini et al., 2011). Recent meta-analyses support the bilateral distribution of activation foci (Bohrn et al., 2012; Rapp et al., 2012). As in the case of the ERP response, familiarity plays an important role also in the localization of the processes (Schmidt and Seger, 2009; Forgács et al., 2012, 2014). EEG data are in line with the bilateral view (Coulson and Van Petten, 2007). Specifically, the N400 effect for metaphors was found to be localized in the bilateral temporal cortex (Arzouan et al., 2007b). In the present study, we run reconstruction of the intracortical ERP origin to further explore the source of the effects, and to compare the results with the previous literature.

## Experiment 1: minimal context

### Methods

#### Participants

Thirteen healthy volunteers (6F; mean age = 25.92, SD = 3.75) took part in the study. All participants were monolingual native speakers of Italian. They were all undergraduate or graduate students with a medium-high educational level (16 years of schooling on average). All participants were right handed. Handedness preference was tested with the 10-item version of the Edinburgh Handedness Inventory (Oldfield, 1971). Participants had an average laterality quotient of 87 (of 100 for complete right-handedness; range 71–100). All participants had normal or corrected-to-normal vision, and reported no serious psychological or physical health problems. The experimental protocol was approved by the local ethical committee and was performed in accordance with the declaration of Helsinki. All participants gave written informed consent.

#### Stimuli

Stimuli were constructed by expanding the set used in a previous neuroimaging study on metaphor comprehension (Bambini et al., 2011). Sixty-four nouns functioned as target words (e.g., “squalo,” *shark*). Nouns were matched for the main psycholinguistic variables, i.e., frequency, word length, orthographic difficulty. Each noun was associated to two other nouns, once literally (e.g., “squalo”-“pesce,” tr. *shark-fish*) once metaphorically (“squalo”-“avvocato,” tr. *shark-lawyer*). Pairs were embedded into two-sentence passages with a minimal context, e.g., literal “Sai che cos'è quel pesce? Uno squalo.” (tr. *Do you know what that fish is? A shark*.) vs. metaphor “Sai che cos'è quell'avvocato? Uno squalo.” (tr. *Do you know what that lawyer is? A shark*.), for a total of 128 passages (64 metaphorical, 64 literal). This passage structure was chosen in order to have an equal number of words in Experiment 1 and Experiment 2 (see below).

All selected metaphors were non-lexicalized, i.e., they were not listed as idiomatic expressions of Italian. However, given the important role of familiarity in processing metaphor in general, it is possible that, even for non-lexicalized metaphors, there are differences in perceived frequency, with impact on ERP patterns. For this reason, we decided to treat familiarity as a possible confounding variable and to control for the familiarity of the metaphorical expressions. To this purpose, we divided the metaphorical set in familiar and non-familiar metaphors, based on a pre-test run on 16 participants matched for age and education with the participants of the ERP study. Participants were presented with a list of metaphors and had to classify each of them as either familiar or non-familiar. Of the 64 metaphorical passages used in this study, 32 were judged as familiar (average agreement 0.78) and 32 were judged as non-familiar (average agreement 0.76). In analyzing behavioral and EEG data, familiar and non-familiar metaphors will be compared preliminary to the main metaphor vs. literal comparison, to control for the presence of effects related to familiarity.

Cloze probability was also pre-tested through a completion (“cloze”) test on a sample of 15 participants matched for age and education with the participants of the ERP study. The noun pairs used to build the two-sentence passages were presented in a single sentence form, truncated before the last word (e.g., for the literal condition, *That fish is a…* and, for the metaphorical condition, *That lawyer is a…*). Mean cloze probability was 0.11 (SD = 0.17) for literal endings and 0.01 (SD = 0.03) for metaphorical endings, with a significant difference between the two conditions (paired *t*-test, *p* < 0.001).

Passages were divided in two lists so that each participant saw a target noun only once, either in the literal or in the metaphorical condition. In addition, 32 filler passages per list were included, containing literal passages of comparable structure.

#### Task

Metaphor comprehension was given as an implicit task and participants were not informed about the presence of metaphors in the stimuli. In order to maintain attention, participants were explicitly instructed to perform an adjective matching task following the comprehension of the target stimuli. Two adjectives were presented after each passage, one on the right, the other on the left of the screen, one on-topic with respect to the preceding passage, the other off-topic. Participants were instructed to select the adjective that better matched with the preceding passage, by pressing the button in their right or left hand. For each pair of passages (literal and metaphorical, split in the two lists), the same adjective pair was used and, so that the materials employed in the task was constant across condition (e.g., for the metaphorical and the literal passages built upon the noun “shark”, the adjective pair was “feroce”, tr. *ferocious*, vs. “geografico”, tr. *geographical*).

#### Procedure

During EEG recording, participants were comfortably seated in a dimly lit sound-attenuated room while stimuli were presented in binocular vision on a video monitor at a viewing distance of about 80 cm. Written stimuli were presented in lowercase white font on a dark background. The task sequence was controlled by a PC running Presentations software (Neurobehavioral Systems, http://www.neurobehavioralsystems.com). Each trial started with a fixation cross presented for 500 ms in the center of the screen, followed by the first part of the passage for 1300 ms. A pre-test showed that this time was sufficient to read and understand the passage. Then, the determiner and the target noun were presented, one at a time for 400 ms each, preceded by 400 ms of blank screen in both conditions (metaphorical and literal). Target nouns were presented together with a dot to indicate the end of the passage. Next, the screen remained black for 1500 ms, and then the adjective pair appeared, with up to 2500 ms allocated for response. The buttons used to indicate the correct adjective (left or right hand) were counterbalanced across subjects. Thereafter, the screen remained blank until the next trial, resulting in a total trial duration of 9200 ms.

Response time (RT) and response accuracy (percentage of correct responses) in the explicit task following the presentation of the target stimuli were recorded. In a preliminary analysis, RTs of the metaphors were submitted to a one-way ANOVA with Familiarity as the independent factor (2 levels, familiar vs. non-familiar). Next, RTs were submitted to a one-way ANOVA with Metaphoricity as the independent factor (2 levels, metaphor vs. literal). Accuracy data were analyzed non-parametrically. First, familiar vs. non-familiar metaphors were analyzed through Wilcoxon signed-rank test; next the same test was used to compare metaphor vs. literal stimuli. The overall alpha level was fixed at 0.05.

#### Electrophysiological recording and analysis

EEG was recorded using BrainVisionTM system with 64 electrodes referenced to the left mastoid (Di Russo and Pitzalis, 2014). Horizontal eye movements were monitored with a bipolar recording from electrodes at the left and right outer canthi. Blinks and vertical eye movements were recorded with an electrode below the left eye, which was referenced to site Fp1. Electrode impedances were kept below 5 kΩ. The EEG from each electrode site was digitized at 250 Hz with an amplifier band-pass of 0.01–60 Hz including a 50 Hz notch filter and was stored for off-line averaging. The EEG was segmented for each target stimulus giving epochs of 1000 ms (from −200 to +800 ms relative to the target noun). Computerized artifacts rejection was performed prior to signal averaging in order to discard epochs in which deviations in eye position, blinks, or amplifier blocking occurred. On average, 6.5% of the trials were rejected. Blinks were the most frequent cause of rejection. ERPs were averaged separately according to the conditions (metaphor vs. literal) with respect to a 100 ms pre-stimulus baseline (in both conditions). To further reduce high-frequency noise, the averaged ERPs were filtered at 30 Hz.

All statistical analyses were performed on the mean ERP amplitudes in the different experimental conditions. On the basis of previous studies on the N400 and P600 in similar contexts (Arzouan et al., 2007a; De Grauwe et al., 2010) and visual inspection of the spatiotemporal ERP patterns, we defined two different time windows (320–440 ms for the N400 and 550–700 ms for the P600) and 25 electrodes (see Table 1) that were submitted to two analyses. In a preliminary one-way ANOVA of the ERP amplitudes for the metaphor condition, performed on each electrode site and each time window, Familiarity was the independent variable (2 levels, familiar vs. non-familiar). Next, for each electrode site and each time window, a one-way ANOVA was performed on all items, with Metaphoricity as the independent factor (2 levels, metaphor vs. literal), adjusting for nonsphericity with the Greenhouse-Geiser epsilon coefficient. In all conditions *t* = 0 ms marked the onset of the target word. The overall alpha level was fixed at 0.05.

**Table 1:** **Experiment 1 (minimal context)**.

**Channel**	**320–440 ms**					**550–700 ms**				
	**M (μV)**	**L (μV)**	***F*-value**	***p***	**$\eta_{\text{p}}^{2}$**	**M (μV)**	**L (μV)**	***F*-value**	***p***	**$\eta_{\text{p}}^{2}$**
F3	−1.869	−1.159	3.050	0.106	0.20	0.010	−0.491	0.775	0.396	0.06
Fz	−1.892	−0.719	6.395	0.026^*^	0.35	0.247	−0.113	0.423	0.528	0.03
F4	−1.945	−0.811	5.751	0.034^*^	0.32	0.177	−0.180	0.643	0.438	0.05
FC1	−1.990	−0.904	5.137	0.043^*^	0.30	0.809	0.074	1.490	0.246	0.11
FCz	−2.115	−0.714	7.312	0.019^*^	0.38	0.818	0.278	0.745	0.405	0.06
FC2	−2.104	−0.691	8.141	0.015^*^	0.40	0.721	0.303	0.584	0.459	0.05
C3	−1.811	−0.795	5.761	0.034^*^	0.32	0.526	0.108	0.736	0.408	0.06
C1	−2.018	−0.917	6.359	0.027^*^	0.35	0.922	0.170	1.844	0.199	0.13
Cz	−2.174	−0.985	5.550	0.036^*^	0.32	1.199	0.280	1.942	0.189	0.14
C2	−1.955	−0.837	5.946	0.031^*^	0.33	1.053	0.189	2.207	0.163	0.16
C4	−1.666	−0.564	5.630	0.035^*^	0.32	0.800	0.139	1.377	0.263	0.10
CP3	−1.408	−0.533	4.665	0.052	0.28	0.702	−0.153	4.504	0.055	0.27
CP1	−1.600	−0.603	4.489	0.056	0.27	0.979	−0.036	3.305	0.094	0.22
CP2	−1.474	−0.483	4.749	0.050^*^	0.28	1.166	0.076	2.752	0.123	0.19
CP4	−1.385	−0.261	5.282	0.040^*^	0.31	0.758	−0.065	1.954	0.188	0.14
P3	−0.757	−0.074	2.762	0.122	0.19	0.887	−0.175	6.836	0.023^*^	0.36
P1	−0.980	−0.245	2.487	0.141	0.17	1.164	−0.140	6.495	0.026^*^	0.35
Pz	−1.175	−0.281	3.141	0.102	0.21	1.253	−0.115	4.940	0.046^*^	0.29
P2	−1.063	−0.302	1.978	0.185	0.14	1.029	−0.294	4.100	0.066	0.25
P4	−1.089	−0.228	2.699	0.126	0.18	0.683	−0.393	2.817	0.119	0.19
PO3	−0.342	−0.114	0.291	0.600	0.02	0.802	−0.415	9.250	0.010^*^	0.44
PO1	−0.648	−0.162	1.183	0.298	0.09	0.950	−0.368	7.939	0.016^*^	0.40
POz	−0.788	−0.305	0.831	0.380	0.06	1.043	−0.435	7.960	0.015^*^	0.40
PO2	−0.946	−0.265	1.644	0.224	0.12	0.750	−0.572	4.294	0.060	0.26
PO4	−1.113	−0.663	0.783	0.394	0.06	0.524	−0.839	5.116	0.043^*^	0.30

Mean amplitude (μV) for the metaphorical (M) and literal (L) conditions in the N400 and P600 time windows on a sample of relevant electrodes, with significance values for the Metaphoricity factor [F_(1, 12)_;

* *p < 0.05]*.

In order to preclude that one or two subjects are influencing the results excessively, we performed a sensitivity analysis, by comparing the previous results with those obtained by deleting randomly two participants two times.

Tridimensional topographical maps and estimation of intracranial sources generating effects on the N400 and the P600 was carried out using the BESA 2000 software (MEGIS Software GmbH, Gräfelfing, Germany). We used the spatiotemporal source analysis of BESA that estimates location, orientation, and time course of equivalent dipolar sources by calculating the scalp distribution obtained for a given model (forward solution). This distribution was then compared to that of the actual ERP. Interactive changes in source location and orientation lead to minimization of residual variance between the model and the observed spatiotemporal ERP distribution. The three-dimensional coordinates of each dipole in the BESA model were determined with respect to the Talairach axes. In these calculations, BESA assumed a realistic approximation of the head (based on the MRI of 24 subjects). The possibility of interacting dipoles was reduced by selecting solutions with relatively low dipole moments with the aid of an “energy” constraint (weighted 20% in the compound cost function, as opposed to 80% for the residual variance). The optimal set of parameters was found in an iterative manner by searching for a minimum in the compound cost function. Latency ranges for fitting were chosen (see above) to minimize overlap between the two, topographically distinctive components. The accuracy of the source model was evaluated by measuring its residual variance as a percentage of the signal variance, as described by the model, and by applying residual orthogonality tests (ROT) (Böcker et al., 1994). The resulting individual time series for the dipole moments (the source waves) were subjected to an orthogonality test, referred to as a source wave orthogonality test (SOT) (Böcker et al., 1994). For all t-statistics, the alpha level was fixed at 0.05.

In order to further explore possible confounding effects of familiarity, we performed an additional analysis with the three conditions (non-familiar metaphors, familiar metaphors, literal). For the same time windows and the same electrode sites used in the main analysis above (metaphor vs. literal), a one-way ANOVA with 3 levels for the Metaphoricity factor was run (non-familiar metaphors, familiar metaphors, literal). Two planned contrasts were run, one between non-familiar and familiar metaphors, and one between the two metaphorical conditions together (familiar metaphors + non-familiar metaphors) and the literal condition.

### Results

#### Behavioral results

Within the metaphor set, RTs in the adjective matching task for familiar and non-familiar metaphors, respectively 1087 ms (SDOM ± 27) and 1098 ms (SDOM ± 26), did not differ significantly [*F*_(1, 17)_ = 0.015; *p* = 0.904], which legitimated pooling together the two conditions. Globally, RTs were 1093 ms (SDOM ± 19) for the metaphor condition and 1091 ms (SDOM ± 18) for the literal condition, with no statistically significant differences [*F*_(1, 17)_ = 0.008; *p* = 0.930). Accuracy did not significantly vary for familiar and non-familiar metaphors, respectively 92.50 and 90.63% (Wilcoxon's *z* = −0.359, *p* = 0.719), which legitimated pooling together the two conditions. Accuracy was high in both condition (91.56% for metaphor and 90.94% for literal condition), with no statistically significant differences (Wilcoxon's *z* = −0.178, *p* = 0.859).

#### EEG results

**Figure 1:**
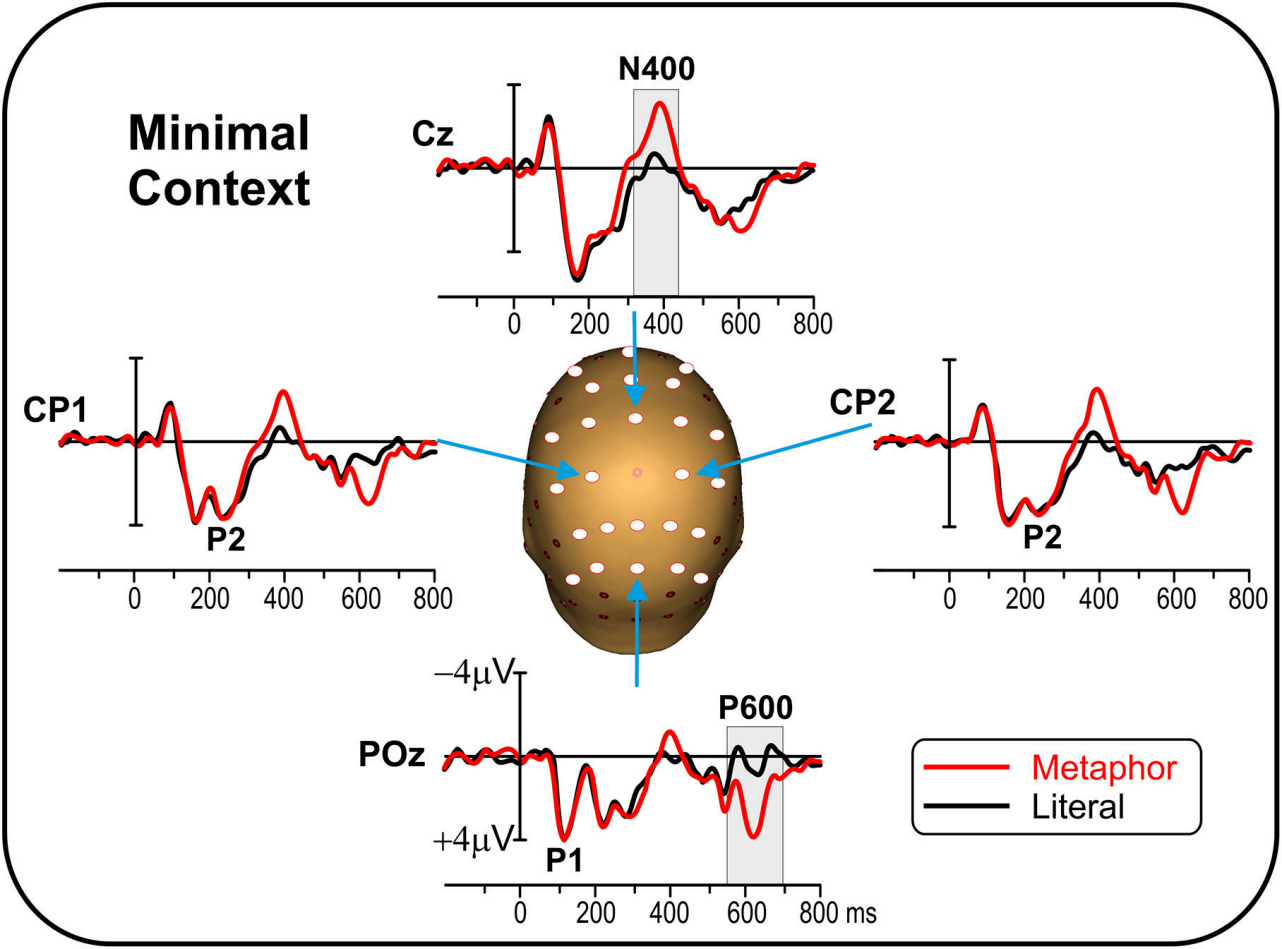
**Experiment 1 (minimal context): Grand average ERP waveforms for metaphor and literal conditions on representative electrodes**. The main ERP components are labeled. Gray bars indicate the time windows used for statistical analysis. Circles indicate the 25 electrode sites used in the analysis.

Figure 1 shows the grand-average ERP for the metaphor and literal conditions over representative electrodes. The earliest detectable ERP component was the visual P1 over bilateral parieto-occipital areas peaking at about 110 ms. The N1 component peaked at about 170 ms over lateral parieto-occipital areas (not shown). The P2 component peaked at about 240 ms over bilateral central-parietal areas. The N400 peaked at about 390 ms over medial central areas and the P600 peaked at about 620 ms over medial parietal areas. The early sensorial components (P1, N1, and P2) were identical in the two conditions, but starting from 300 ms the two waveforms started to diverge showing larger N400 and P600 for the metaphor condition. The shaded gray areas indicate the time windows used for statistical analyses.

The preliminary ANOVA for the Familiarity factor did not yield any significant result on any of the electrodes considered, in any of the two time windows (all *ps* > 0.05), which legitimated pooling together the two metaphorical conditions (familiar and non-familiar metaphors) and comparing with the literal condition. The main ANOVA between the metaphor and the literal conditions revealed a significant effect of condition in the N400 time window on fronto-central, central, and centro-parietal sites. The main ANOVA also revealed a significant effect of condition in the P600 time window on parietal and parieto-occipital sites. See Table 1 for mean amplitudes and significance values.

The sensitivity analysis yielded the same effects, i.e., an N400 effect over fronto-central, central and centro-parietal sites, and a P600 over parietal and parieto-occipital sites. See Supplementary Tables 1.1 and 1.2 in Supplemental Data Sheet.

**Figure 2:**
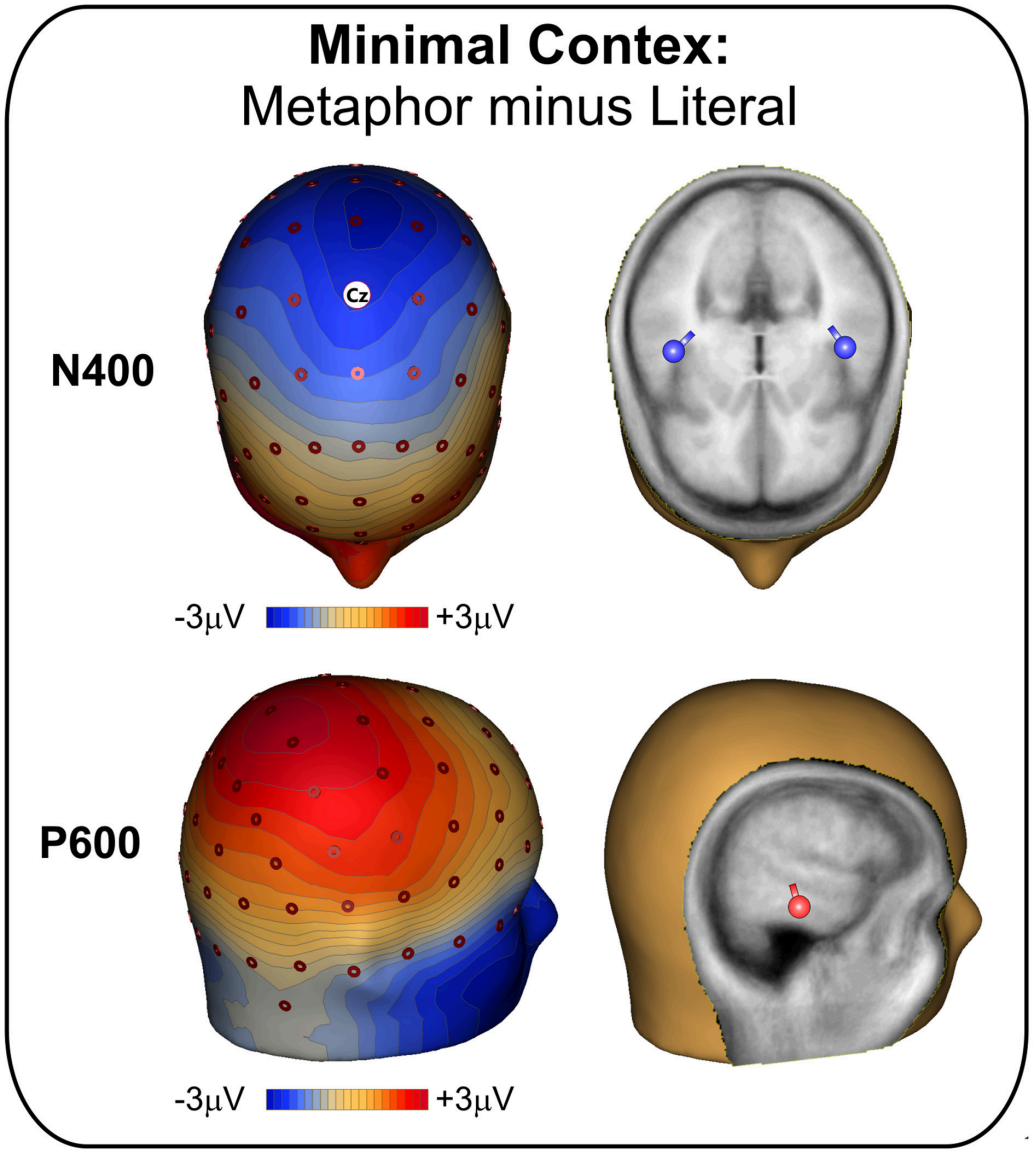
**Experiment 1 (minimal context): Topographic scalp distribution of the N400 and P600 effects (left) and their intracranial source localization rendered in a realistic head template**.

Figure 2 shows the scalp topography of the differential ERP waveform obtained from the subtraction of the metaphor minus the literal condition. The N400 effect had a medial central distribution spreading over the two hemispheres, which was bilaterally localized within the superior temporal lobe (BA 22). The P600 effect had a medial parietal distributions more spread out over the right hemisphere, which was localized in the right inferior temporal lobe (BA 20).

The additional analysis with the three conditions (non-familiar metaphors, familiar metaphors, literal) confirmed the main analysis. A main effect was visible in the N400 time window on fronto-central, central and centro-parietal electrodes and in the P600 windows on parieto-occipital sites. Planned contrasts showed that the effect is triggered by the comparison between the two metaphorical conditions together (familiar + non-familiar) vs. the literal condition, rather than by familiarity. See Supplementary Tables 2.1 and 2.2 in Supplemental Data Sheet.

### Discussion

Behavioral data showed that participants easily performed the adjective matching task, with no differences between literal and metaphorical stimuli. This is in line with previous studies employing the same task (Bambini et al., 2011) and suggests that subjects correctly processed the passages. The comparison between the ERP waveforms for literally and metaphorically used words in minimal context showed a biphasic pattern, with higher N400 and P600 amplitudes for metaphors as compared to literal stimuli. These findings were confirmed by the sensitivity analysis. The biphasic pattern observed here is compatible with previous studies employing metaphors in the “A is B” form and in minimal context (De Grauwe et al., 2010; Weiland et al., 2014). The topography of the N400 shows bilateral distribution over centro-medial sites, localized within the superior temporal lobes, in line with previous studies on the N400 in general (Van Petten and Luka, 2006; Kutas and Federmeier, 2011). The P600 appears more spread out on the right hemisphere, localized within the right inferior temporal lobe. The topographic distribution of the P600/LPC is still a matter of debate over the literature (Arzouan et al., 2007a; Yang et al., 2013). Supporting evidence for our data can be found in fMRI data on metaphor processing on the same materials (Bambini et al., 2011), pointing to a greater involvement of right temporal areas.

We also reported the absence of effects related to familiarity, both in the preliminary analysis comparing familiar and non-familiar metaphors, and in the analysis on all items. On the one hand this supports the idea that the biphasic pattern observed for metaphor was not affected by confounding effects due to familiarity. On the other hand this might seem in contrast with previous literature reporting strong familiarity modulation of the ERP response (Arzouan et al., 2007a; Lai et al., 2009). However, this discrepancy might be explained by noting that previous studies contrasted highly conventional and highly novel metaphors, while in our study all metaphors are non-lexicalized, although associated with different judgments of familiarity in the pre-test.

Although both the N400 and the P600 effects reflect pragmatic processing, in this first experiment it is not possible to specifically weigh the role of context, as it might shape the whole process of metaphor comprehension. In order to disentangle the role of context, we conducted a second experiment, embedding the target words in supportive linguistic information, expressing the ground between the metaphor's topic and vehicle.

## Experiment 2: supportive context

### Methods

#### Participants

Thirteen healthy volunteers (7F; mean age = 26.00 years, SD 3.70) took part in the study. All participants were monolingual native speakers of Italian. They were all undergraduate or graduate students with a medium-high educational level (16 years of schooling on average). All participants were right handed. Handedness preference was tested with the 10-item version of the Edinburgh Handedness Inventory (Oldfield, 1971). Participants had an average laterality quotient of 86 (of 100 for complete right-handedness; range 69–100). All participants had normal or corrected-to-normal vision, and reported no serious psychological or physical health problems. The experimental protocol was approved by the local ethical committee and was performed in accordance with the declaration of Helsinki. All participants gave written informed consent.

#### Stimuli

The same 64 noun pairs employed in Experiment 1 were used and embedded in a supportive context. Pairs (e.g., *shark-fish* and *shark-lawyer*) were inserted into two-sentence passages where the link between the noun and its associate was made explicit. In the case of metaphor, this corresponded to the so-called ground, i.e., the property the bonds metaphor's topic and metaphor's vehicle. The structure of the passages was such that the overall number of words did not vary with respect to Experiment 1 (i.e., 8 words). Literal passages were of the type: “Quel pesce è molto aggressivo. È uno squalo.” (tr. *That fish is really aggressive. It is a shark*.) and metaphorical passages were of the type: “Quell'avvocato è molto aggressivo. È uno squalo.” (tr. *That lawyer is really aggressive. He is a shark*.), for a total of 128 passages. A pre-test of cloze probability was run on 14 participants matched for age and education to the participants of the ERP, by showing the literal and the metaphorical passages truncated before the last word (e.g., for the literal condition, *That fish is really aggressive. It is a…* and, for the metaphorical condition, *That lawyer is really aggressive. He is a…*). Cloze probability was 0.35 (SD = 0.28) for literal passages and 0.12 (SD = 0.16) for metaphorical passages, with a significant difference between the two conditions (paired *t*-test, *p* < 0.001). Although still in the range of values classified as low contextual constraint (Kutas and Hillyard, 1984), these cloze probability values differed significantly from the values for the literal and metaphorical endings in Experiment 1 (paired *t*-test, *p* < 0.001). This shows that adding the link between the noun and its associate successfully increased context-based expectations both for literal and metaphorical conditions in Experiment 2. Passages were divided in two lists so that each participant saw a target noun only once. In addition, 32 filler passages per list were included, containing literal passages of comparable structure.

#### Task

As in Experiment 1, participants were asked to perform an adjective matching task following the presentation of the target stimuli.

#### Procedure

The same as for Experiment 1.

#### Electrophysiological recording and analysis

Data were recorded as in Experiment 1. For the analysis of the ERP component, we used the same time windows selected in Experiment 1 (320–440 and 550–700 ms) to allow for the comparison of the results. As for Experiment 1, we conducted a preliminary ANOVA for Familiarity (familiar vs. non-familiar metaphors), a main ANOVA (metaphor vs. literal conditions) and a source analysis. Likewise, we also performed a sensitivity analysis and an additional analysis with the three conditions (non-familiar metaphors, familiar metaphors, literal).

Moreover, in order to directly compare the findings of Experiment 1 and 2 we conducted an additional cross-experiment analysis including Context as a between participants factor. For a similar approach see Tune et al. (2014).

### Results

#### Behavioral results

RTs in the adjective matching task for familiar metaphors (1044 ms, SDOM ± 22) and non-familiar metaphors (1043 ms, SDOM ± 22) did not differ significantly [*F*_(1, 18)_ = 0.000; *p* = 0.986], which legitimated pooling together the two conditions. RTs was 1044 ms (SDOM ± 16) for the metaphor condition and 1037 ms (SDOM ± 17) for the literal condition, with no significant differences [*F*_(1, 18)_ = 0.008; *p* = 0.982]. Accuracy was 97.50% for familiar metaphors and 96.88% for non-familiar metaphors, with no significant differences (*z* = −0.333, *p* = 0.739), which legitimated pooling together the two conditions. Accuracy was 97.19% for metaphors and 95.31% for the literal condition, with no statistically significant differences (*z* = −1.403, *p* = 0.161).

#### EEG results

**Figure 3:**
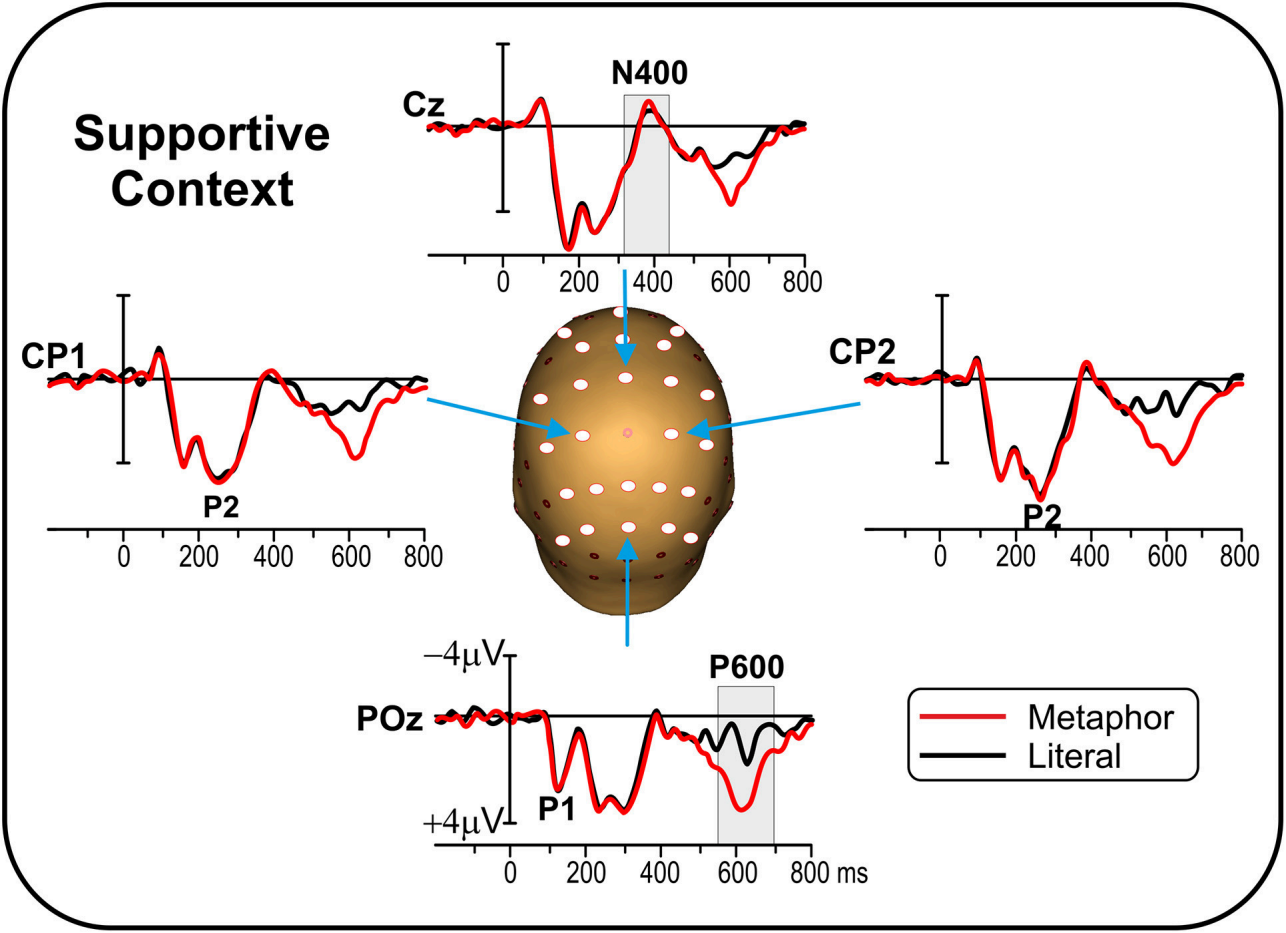
**Experiment 2 (supportive context): Grand average ERP waveforms for metaphor and literal conditions on representative electrodes**. The main ERP components are labeled. Gray bars indicate the time windows used for statistical analysis. Circles indicate the 25 electrode sites used in the analysis.

Figure 3 shows the grand-average ERP for the metaphor and literal conditions over representative electrodes. The ERP components were similar as in the Experiment 1 except for the N400, which was almost the same in the two conditions (metaphorical and literal). The shaded gray areas indicate the time windows used for statistical analyses.

The preliminary ANOVA on Familiarity did not yield any significant effect on any electrode site in any time window (all *p* > 0.05), which legitimated pooling together familiar and non-familiar metaphors. In the main ANOVA on Metaphoricity (metaphor vs. literal), no significant effects were observed in the N400 time window. On the contrary, in the P600 time window ANOVA yielded a significant effect of Metaphoricity on frontal, central, and parietal electrodes. See Table 2 for mean amplitudes and significance values. The sensitivity analysis yielded the same results, with no N400 effects and a P600 effects on frontal, central and parietal electrodes. See Supplementary Tables 1.3 and 1.4 in Supplemental Data Sheet.

**Table 2:** **Experiment 2 (supportive context)**.

**Channel**	**320–440 ms**					**550–700 ms**				
	**M (μV)**	**L (μV)**	***F*-value**	***p***	**$\eta_{\text{p}}^{2}$**	**M (μV)**	**L (μV)**	***F*-value**	***p***	**$\eta_{\text{p}}^{2}$**
F3	−0.986	−1.475	0.944	0.350	0.07	0.353	−1.031	5.896	0.032^*^	0.33
Fz	−0.789	−0.907	0.046	0.833	0.00	0.629	−1.025	11.171	0.006^**^	0.48
F4	−1.042	−0.664	0.487	0.499	0.04	0.282	−1.026	7.074	0.021^*^	0.37
FC1	−0.870	−0.770	0.029	0.868	0.00	1.150	−0.422	8.800	0.012^*^	0.42
FCz	−0.809	−0.589	0.140	0.715	0.01	1.175	−0.461	12.707	0.004^**^	0.51
FC2	−0.993	−0.462	0.824	0.382	0.06	0.994	−0.472	11.553	0.005^**^	0.49
C3	−0.548	−0.336	0.211	0.654	0.02	1.061	−0.095	6.280	0.028^*^	0.34
C1	−0.777	−0.387	0.472	0.505	0.04	1.224	−0.069	7.933	0.016^*^	0.40
Cz	−0.958	−0.408	0.685	0.424	0.05	1.349	−0.084	10.059	0.008^**^	0.46
C2	−0.862	−0.189	1.173	0.300	0.09	1.233	−0.124	7.752	0.017^*^	0.39
C4	−0.721	−0.047	1.487	0.246	0.11	0.980	−0.298	7.109	0.021^*^	0.37
CP3	−0.051	0.167	0.238	0.635	0.02	1.100	0.004	5.890	0.032^*^	0.33
CP1	−0.266	0.183	0.656	0.434	0.05	1.156	0.016	5.358	0.039^*^	0.31
CP2	−0.425	0.174	1.205	0.294	0.09	1.166	−0.186	8.501	0.013^*^	0.41
CP4	−0.345	0.190	1.408	0.258	0.11	1.057	−0.490	10.628	0.007^**^	0.47
P3	0.361	0.668	0.691	0.422	0.05	1.102	−0.048	7.200	0.020^*^	0.37
P1	0.212	0.622	0.850	0.375	0.07	1.108	0.039	4.644	0.052	0.28
Pz	0.029	0.677	1.849	0.199	0.13	1.164	−0.025	5.727	0.034^*^	0.32
P2	0.028	0.482	1.037	0.329	0.08	1.069	−0.293	5.821	0.033^*^	0.33
P4	−0.113	0.445	1.457	0.251	0.11	0.900	−0.514	6.252	0.028^*^	0.34
PO3	0.371	0.857	2.273	0.158	0.16	0.887	0.141	2.134	0.170	0.15
PO1	0.129	0.533	0.834	0.379	0.07	0.880	−0.100	2.340	0.152	0.16
POz	0.208	0.875	3.049	0.106	0.20	1.087	−0.011	3.589	0.083	0.23
PO2	0.061	0.643	1.992	0.184	0.14	0.908	−0.298	3.514	0.085	0.23
PO4	−0.366	0.192	2.169	0.167	0.15	0.566	−0.515	2.737	0.124	0.19

Mean amplitude (μV) for the metaphorical (M) and literal (L) conditions in the N400 and P600 time windows on a sample of relevant electrodes, with significance values for the Metaphoricity factor [F_(1, 12)_;

* p < 0.05;

** *p < 0.01]*.

**Figure 4:**
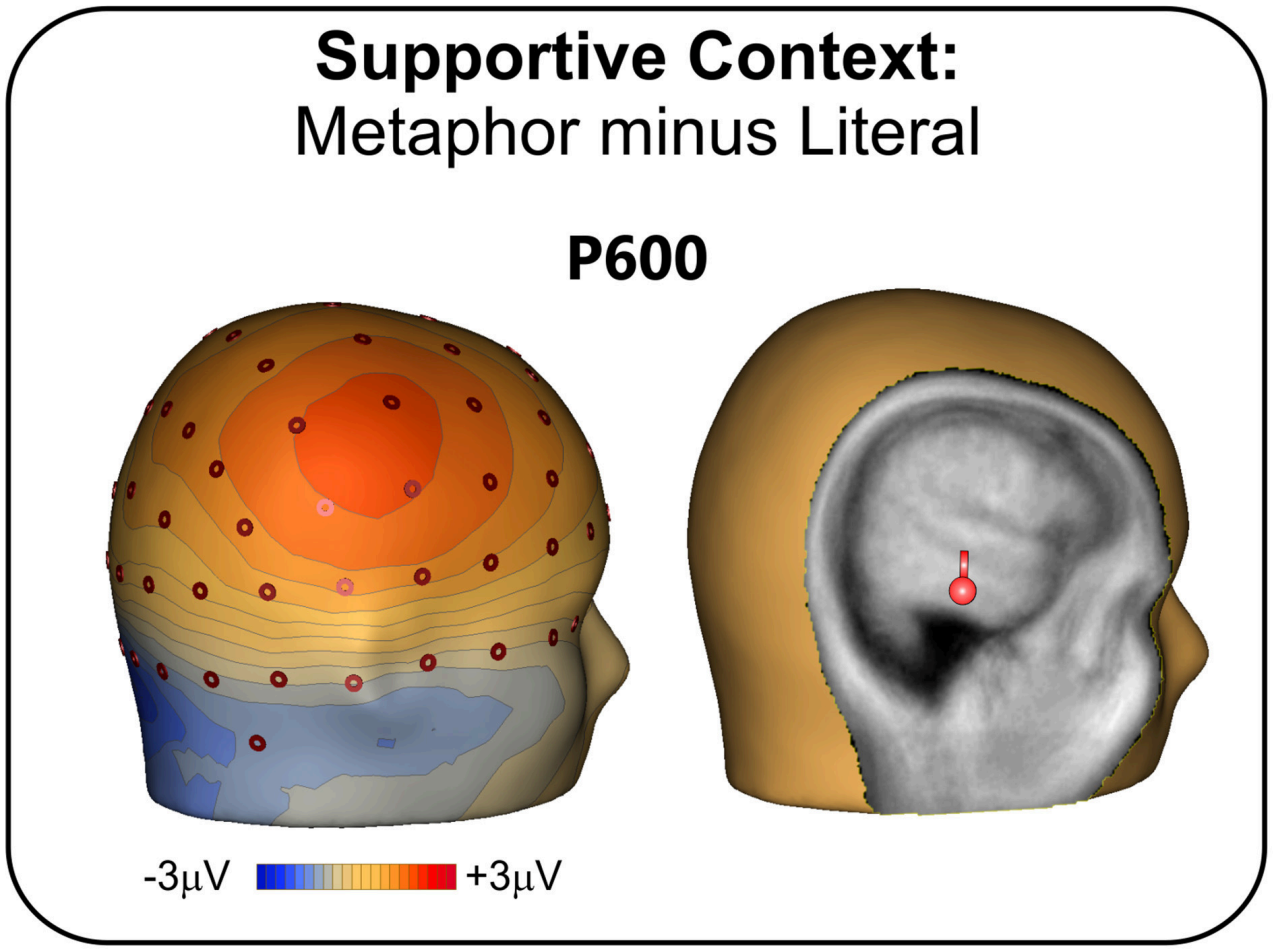
**Experiment 2 (supportive context): Topographic scalp distribution of the P600 effect (left) and its intracranial source localization rendered in a realistic head template**.

Figure 4 shows the scalp topography of the differential ERP waveform obtained from the subtraction of the metaphor minus the literal condition. The P600 effect had a clearly parietal distribution over the right hemisphere, and was localized in the right inferior temporal lobe (BA 20) similarly to the P600 localization in Experiment 1.

The additional analysis with the three conditions (non-familiar metaphors, familiar metaphors, literal) confirmed the main analysis. A main effect was visible in the P600 window on frontal, central and parietal sites, and planned contrasts showed that this effect is triggered by the comparison between the two metaphorical conditions considered together (familiar + non-familiar) vs. the literal condition. In the N400 window, only a few right posterior electrodes showed a main effect, possibly due to familiarity. See Supplementary Tables 2.3 and 2.4 in Supplemental Data Sheet.

In the N400 time window, the cross-experiment analysis showed an interaction between Context and Metaphoricity on right central and parietal sites [C4 $F_{\left(2,\;48\right)}=3.269,p=0.047,\eta_{\text{p}}^{2}=0.12$; C6 $F_{\left(2,\;48\right)}=3.974,p=0.025,\eta_{\text{p}}^{2}=0.14$; CP4 $F_{\left(2,48\right)}=3.321,p=0.045,\eta_{\text{p}}^{2}=0.12$; CP6 $F_{\left(2,\;48\right)}=3.987,p=0.025,\eta_{\text{p}}^{2}=0.14$; FC6 $F_{\left(2,\;48\right)}=3.334,p=0.044,\eta_{\text{p}}^{2}=0.12$; I6 $F_{\left(2,\;48\right)}=4.037,p=0.024,\eta_{\text{p}}^{2}=0.14$; P4 $F_{\left(2,\;48\right)}=3.652,p=0.033,\eta_{\text{p}}^{2}=0.13$; P6 $F_{\left(2,\;48\right)}=4.643,p=0.014,\eta_{\text{p}}^{2}=0.16$; P8 $F_{\left(2,\;48\right)}=4.626,p=0.015,\eta_{\text{p}}^{2}=0.16$; PO8 $F_{\left(2,\;48\right)}=4.070,p=0.023,\eta_{\text{p}}^{2}=0.14$; T8 $F_{\left(2,\;48\right)}=9.511,p=0.000,\eta_{\text{p}}^{2}=0.28$; TP8 $F_{\left(2,\;48\right)}=5.839,p=0.005,\eta_{\text{p}}^{2}=0.20$]. In the P600 window, there is a significant interaction between Context and Metaphoricity only on Fp1 [*F*_(2, 48)_ = 4.188, *p* = 0.021, $\eta_{\text{p}}^{2}$ = 0.15]. In other words, the analysis of the N400 time window showed an effect of the between participants factor on right posterior sites: the N400 effect for metaphor was bigger without a supportive context (Experiment 1) than with a supportive context (Experiment 2).

### Discussion

As in Experiment 1, behavioral data showed that participants correctly processed the stimuli, with no differences between metaphors and literal stimuli. The ERP responses, time-locked to the target words, varied across conditions, with enhanced P600 for metaphorical as compared to literal stimuli, confirmed in the sensitivity analysis. The P600 response had a broader distribution than in Experiment 1 but it was localized in the right temporal lobe as in Experiment 1. Notably, in contrast with Experiment 1, there was no N400 effect. These data seem to suggest that context manipulation has a direct impact on the N400, while not suppressing the P600. The cross-experiment analysis supports this interpretation, showing the interaction between metaphoricity and context in the N400 time window.

As in Experiment 1, familiarity seemed to play no role, neither in the preliminary analysis nor in the additional analysis on all items. Effects were limited to a few posterior electrodes in the N400 time window in the planned contrasts in the analysis on all items. Although this might suggest a possible modulation of the N400 response linked to familiarity, this result, however, is too limited to draw further conclusions. It is indeed likely that familiarity becomes evident in the electrophysiological response only over a certain threshold of difference across stimuli, as in previous studies where highly conventional and highly novel metaphors were compared. In contrast, here all metaphorical stimuli consisted of non-lexicalized metaphors, although with different judgments of familiarity in the pre-test.

In what follows the discussion will concentrate on the N400 and the P600 for metaphor, as differently modulated across the two experiments.

## General discussion

The primary aim of this study was to assess the role of context in metaphor comprehension. Results showed the presence of a biphasic N400-P600 pattern when metaphors were presented in a minimal context (Experiment 1). Crucially, when sentences were preceded by a supportive context, the biphasic pattern was not maintained, with metaphors evoking only a P600 effect (Experiment 2). The data obtained for metaphor in minimal context are in line with previous literature. The novel finding here is represented by the effect of the addition of supportive contextual material, which determined the suppression of the N400 effect, but did not suppress the P600 for metaphors. This paves the way to a number of considerations related to the functional characteristics of the observed ERP components.

With respect to the N400, the results of the two experiments suggest that this negativity is especially sensitive to the contextual aspects of pragmatic processing. This hypothesis is also supported by the cross-experiment analysis, which evidenced that the N400 effect for metaphor was bigger without a supportive context (Experiment 1) than with a supportive context (Experiment 2). Previous studies are consistent in reporting enhanced N400 amplitude for metaphorical compared to literal sentences in minimal context or word pairs (Pynte et al., 1996; Tartter et al., 2002; Lai et al., 2009; De Grauwe et al., 2010), yet vary in interpreting its functional role. Here we show that this effect is probably linked to efforts related to the absence of a supportive context, when expectations about upcoming words are not matched.

It is important to highlight that context in our experiment consists of linguistic material constituting the ground, i.e., a property of the lexical concept expressed by the metaphor's vehicle that is promoted and applied to the metaphor's topic. Adding the ground resulted in higher cloze probability rates for metaphorical expressions in Experiment 2 than in Experiment 1, as shown in the pre-test. Given that cloze probability is usually considered a measure of the degree to which the context establishes an expectation for a particular upcoming word (Kutas and Federmeier, 2011; Bambini et al., 2014), we can legitimately say that the two experiments vary with respect to contextual support, and it seems likely to assume that the different N400 response is specifically related to contextual expectations that guide lexical access and retrieval. When the ground is explicit in the context, as in Experiment 2, the retrieval of the metaphorically used words is less costly, as part of the concept is already activated. This interpretation of the N400 effect is in line with several sources of data. First, this account was already proposed in a study where lexical priming affected the N400 for metaphor (Weiland et al., 2014): although in that study the prime was a literal property, such property was still part of the lexical concept expressed by the metaphor vehicle, and it reduced the N400. Second, a similar manipulation in metonymic shift produced a suppression of the N400 when the semantic field of the metonymic concept was activated through lexical items in the context (Schumacher, 2014). More generally, context is known to affect the N400, which responds to the manipulation of semantic congruency at the level of both sentence and discourse, as well as extralinguistic context (Federmeier et al., 2007; van Berkum, 2009; Hoeks and Brouwer, 2014). This interpretation is consistent with the lexical pre-activation based view of the N400, where it is assumed that context has “excitatory” power in supporting lexical retrieval. This kind of proposal comes from studies arguing that the N400 reflects the mental processes that accompany the retrieval of lexical information from long-term memory as facilitated by the activation of features in the preceding context (Brouwer et al., 2012). More generally, the N400 might index the activity of a language processor that rapidly recovers information from multiple sources (e.g., syntax, semantics, discourse, world knowledge) to continuously update its interpretation of an incoming sentence (Stroud and Phillips, 2012). Interestingly, converging evidence of the N400 as an index of contextual expectations also comes from a study on 19-month old children where context for words was represented by colored pictures of objects, suggesting that the functional characterization of this component is very strong since early developmental stages (Friedrich and Friederici, 2004).

With respect to the P600, the positivity observed in our experiment seems to index an authentic pragmatic process of establishing the intended meaning of a metaphor. In current pragmatic models, understanding metaphors is indeed the result of a pragmatic inference exploiting world knowledge, the context, and the lexical meaning of the expression (Carston, 2010a; Pouscoulous, 2014). Specifically, metaphorical interpretation can be seen as an inferential move from the literal meaning to the intended meaning, which starts from the premises in the decoded meaning, combines contextual assumptions, and derives a set of conclusions warranted by the premises (Wilson and Carston, 2007)^1^. The P600 response could thus reflect the derivation of the intended meaning, which capitalizes on context beyond the process of lexical access as observed in the N400 response. Evidence in favor of this interpretation comes from several studies. Our supportive context condition can be compared to the probes employed by Yang et al. in a word-to-sentence matching paradigm where metaphors and literal sentences were preceded by differently congruent words. In line with our findings, that study showed a modulation of the P600, with no N400 effects (Yang et al., 2013). Moreover, several other pragmatic phenomena evoke a P600/LPC effect, among which indirect requests (Coulson and Lovett, 2010) and jokes (Coulson and Kutas, 2001). Interestingly, the P600/LPC effect shows up also in the absence of higher amplitude in the N400 time window, as in the case of irony (Regel et al., 2011; Spotorno et al., 2013), ambiguous idioms processing (Canal et al., 2015), question/answer pairs and other aspects of conversation and discourse (Hoeks et al., 2013; Hoeks and Brouwer, 2014). Regel and colleagues discuss this issue for the specific case of irony, arguing that the absence of the N400 effect is motivated by the easy integration of words in context, while the complete understanding of intended meanings still require later additional cognitive processes in the P600 (Regel et al., 2011). Similarly, for metaphor, when lexical access is facilitated by providing enough supporting context, words are easily integrated, but the final interpretation remains more costly than in the literal case. In this view, the P600 might index the step in the pragmatic inferential process when the speaker comes up with an interpretation of the intended meaning of the utterance, which is observed for metaphors, as well as for irony and other pragmatic phenomena.

More generally, the present study gives additional support to the characterization of the P600 as a reflection of processing costs related to the semantic/pragmatic level (Bornkessel-Schlesewsky and Schlesewsky, 2008; Brouwer et al., 2012), overcoming the classic view of the P600 as a syntactic component, as the stimuli employed in our experiments were neither syntactically anomalous nor ambiguous, and did not differ in syntactic structure across conditions. In line with recent proposals, it might be possible that the P600 is not a single component, but actually a family of late positivities that reflect the word-by-word construction, reorganization, or updating of a mental representation of what is being communicated (Hoeks and Brouwer, 2014). Possibly this family of positivities might belong to the wider P3 family. This hypothesis has been recently revived with the description of the P600 as a point in time where a linguistic entity has achieved subjective significance and some form of adaption process is underway (Sassenhagen et al., 2014).

With respect to the classic debate over the direct vs. indirect view, our study does not offer straightforward conclusions, as a simple comprehension task like the one employed here does not allow to assess the presence of a literal stage. However, the modulation of context might shed some light on the processing steps of the comprehension process. Given the data of Experiment 1 and 2, it is clear that the processing of metaphors is more costly (as shown by the N400 and the P600) than the processing of literal language. Moreover, when a metaphor is preceded by a supportive context (Experiment 2), the effort in the lexical access phase is reduced (no N400), but there is still a P600. Although clearly not decisive on its own, these results are compatible with studies arguing for the lingering of literal meaning (Rubio Fernandez, 2007; Carston, 2010b; Weiland et al., 2014): literal meaning aspects are accessible early on and active throughout the lexical retrieval stage reflected in the N400 in Experiment 1. With supportive context as in our Experiment 2, lexical access becomes easier, as aspects of the metaphorical meaning are activated in the ongoing discourse context, with presumably reduced lingering effects and no visible N400 response. The presence of a P600 in both experiments seems to reflect enhanced costs in a later stage of pragmatically driven interpretative processes: the speaker's meaning, even in the case of a supportive context, must be inferentially derived, hence extra processing is required, both in Experiment 1 and Experiment 2.

Taken together, the results of the two experiments presented here suggest three main conclusions: (i) the pragmatic process of metaphor comprehension unfolds through two different stages which might be explained in terms of retrieval of lexical elements shaped by context followed by pragmatic interpretation; (ii) linguistic context reduces the effort in retrieving lexical aspects of metaphors as indexed in the N400, which has never been observed before in the literature; (iii) linguistic context does not suppress later pragmatic interpretation efforts needed in order to derive the speaker's intended meaning, as reflected in the P600. Although these conclusions seem to capture what happens in the comprehension of metaphors in natural conversation, where the linguistic material often introduces and “primes” metaphorical meaning, they cannot be extended to all possible contexts. For metaphors taken from poetry, for instance, there is behavioral evidence that the literary text cannot be simply considered as a context licensing the figurative expression, but rather it seems to promote mechanisms that make the metaphor more open to different interpretations in different scenarios, less familiar but more meaningful (Bambini et al., 2014). Moreover, the modulation of familiarity might affect both the lexical retrieval and the pragmatic interpretation stage, which was not observed here given that all metaphors were non-lexicalized.

As a second aim of the study, we explored the spatial characteristics and the localization of the two ERP effects, in order to add to the large debate in the imaging literature over the neural correlates of metaphor comprehension and the right hemisphere advantage (Bohrn et al., 2012; Rapp et al., 2012). The N400 observed in Experiment 1 has a standard centro-medial distribution. The source localization analysis indicated the bilateral superior temporal cortex (BA22) as the origin of the effect, in line with previous accounts of the N400 in general (Van Petten and Luka, 2006; Kutas and Federmeier, 2011). This also matches with previous ERP evidence on metaphor, obtained with hemi-field presentation (Coulson and Van Petten, 2007) and with source localization analysis (Arzouan et al., 2007b), disconfirming the right hemisphere advantage and supporting the idea that both hemispheres work in tandem in metaphor comprehension. This is also compatible with imaging studies, where BA 22 is involved in the comprehension of metaphor processing, both in the right (Mashal et al., 2005; Bambini et al., 2011) and in the left hemisphere (Rapp et al., 2012).

The results on the P600 are less straightforward. In Experiment 1, the P600 effect has a standard parietal distribution, which becomes broader and extended to frontal sites in Experiment 2. The topographic features of the semantic P600 are still a matter of debate in the literature, which is too modest-size to derive strong conclusions (Van Petten and Luka, 2012; Regel et al., 2014). Our data suggest that the distribution of the positivity might vary based on context, possibly with a more global process and a distributed involvement of scalp sites when context is supportive enough in early phases, and interpretation is concentrated in later stages. Moreover, in both Experiment 1 and 2, the generator of the P600 effect was localized in the right inferior temporal gyrus (BA 20). Although there seems to be some agreement on the localization of the P600 in the left hemisphere (Brouwer and Hoeks, 2013), the literature disagrees with respect to the P600 for metaphor, with some localizing the effect in the left (Yang et al., 2013) and other in the right hemisphere (Arzouan et al., 2007a). Imaging data can shed some light over this conflict. Sometimes reported for figurative language processing (Eviatar and Just, 2006), right BA 20 is a region implicated also in the evaluation of alternative meanings and interpretation of ambiguous stimuli (Zempleni et al., 2007), as well in the attribution of intentions to story characters (Brunet et al., 2000). Once lexical retrieval is passed, the ultimate interpretative effort in understanding a metaphor involves the attribution of communicative intentions. The right-sided effect of the P600 observed in our study might thus be related to the interpretative effort in deriving the speaker's meaning. Granted that modern literature has largely reconsidered the right hemisphere advantage (see above), the rightward asymmetry found in the present study seems in favor, at the least, of a larger contribution of the right hemisphere for what concerns the final, interpretative part of the pragmatic inference process. This localization and this interpretation of the P600, however, needs to be further verified, possibly combining EEG and imaging data. Overall, considering both the N400 and the P600 results, what our data seem to highlight is the role of the temporal cortex, bilaterally, and possibly with a right focus, which is in line with a recent neurofunctional proposal of temporo-parietal circuitry for pragmatic processing, at the interface between linguistic and social cognition processes (Catani and Bambini, 2014).

## Conclusions

Overall, our findings confirm the presence of two dissociable ERP signatures in the processing of metaphors, namely the N400 indexing lexical access guided by contextual expectation, and the P600, indexing a truly pragmatic interpretative mechanism of deriving the speaker's meaning. When the context is supportive, lexical access is facilitated, but the efforts related to establishing a pragmatic interpretation remain. These results shed light on the comprehension of metaphor in natural conversation and points in the direction of increasing the ecological validity of experimental approaches to pragmatics.

## Author contributions

Design and construction of the materials: VB. Data collection: VB, CB, AS. Data analysis: VB, CB, WS, FDR. Manuscript writing: VB, WS, FDR. All authors provided feedback on the draft and approved the final version of the manuscript.

### Conflict of interest statement

The authors declare that the research was conducted in the absence of any commercial or financial relationships that could be construed as a potential conflict of interest.

## Footnotes

^1^In Relevance Theory, the interpretation of a metaphorical utterance consists of a non-demonstrative inference process that combines the lexical meanings and the context of use. For instance, the interpretation of “Sally is a chameleon” takes as input a premise such as “The speaker has said ‘Sally is a chameleon’ (i.e., a sentence with a fragmentary decoded meaning requiring inferential completion and complementation),” together with other contextual assumptions, and yields as output a conclusion such as “The speaker meant that Sally_x_ is a CHAMELEON^*^, Sally_x_ is changeable, Sally_x_ has a capacity to adapt to her surroundings, it's hard to discern Sally_x_'s true nature (etc.),” where CHAMELEON^*^ is an expansion from the category CHAMELEON to the category CHAMELEON^*^, which includes both actual chameleons and people who share with chameleons the encyclopaedic property of having the capacity to change their appearance in order to blend in with their surroundings (Wilson and Carston, 2007). Of course, this interpretative process takes place at risk, given that the premise cannot guarantee the truth of the conclusion. Yet speakers possess an inferential heuristic for constructing the best interpretation given the available evidence in the context of use.
