# Supplementary material for: Disentangling Metaphor from Context: An ERP Study
Source: Front Psychol. 2016 May 3;7:559. doi: 10.3389/fpsyg.2016.00559 (PMC4853386; doi:10.3389/fpsyg.2016.00559)
Supplement: Supplementary file 1 [file DataSheet1.pdf]

Supplementary Material

# *Supplemental Data Sheet: Additional Analyses*

# Disentangling metaphor from context: An ERP study

**Valentina Bambini*, Chiara Bertini, Walter Schaeken, Alessandra Stella, Francesco Di Russo**

*** Correspondence:** Valentina Bambini: valentina.bambini@iusspavia.it

## Supplementary Tables 1. Sensitivity analysis

**Supplementary Table 1.1.** Experiment 1 (minimal context) – Subsample 1 (N=11): Mean amplitude (μV) for the metaphorical (M) and literal (L) conditions in the N400 and P600 time windows on a sample of relevant electrodes, with significance values for the Metaphoricity factor (F (1,10); * p < 0.05; ** p < 0.01).

| | 320-440 ms | | | | | 550-700 ms | | | | |
|---|---|---|---|---|---|---|---|---|---|---|
| Channel | M (μV) | L (μV) | F value | p | $\eta_p^2$ | M (μV) | L (μV) | F value | p | $\eta_p^2$ |
| F3 | -1.447 | -0.902 | 1.501 | 0.249 | 0.13 | 0.048 | -0.461 | 0.590 | 0.460 | 0.06 |
| Fz | -1.490 | -0.400 | 4.965 | 0.050* | 0.33 | 0.198 | -0.104 | 0.230 | 0.642 | 0.02 |
| F4 | -1.596 | -0.436 | 5.002 | 0.049* | 0.33 | 0.125 | -0.333 | 0.800 | 0.392 | 0.07 |
| FC1 | -1.683 | -0.684 | 3.174 | 0.105 | 0.24 | 0.824 | 0.176 | 0.970 | 0.348 | 0.09 |
| FCz | -1.794 | -0.459 | 4.869 | 0.052 | 0.33 | 0.796 | 0.382 | 0.358 | 0.563 | 0.03 |
| FC2 | -1.804 | -0.377 | 5.951 | 0.035* | 0.37 | 0.715 | 0.317 | 0.415 | 0.534 | 0.04 |
| C3 | -1.651 | -0.631 | 4.922 | 0.050* | 0.33 | 0.654 | 0.323 | 0.360 | 0.562 | 0.03 |
| C1 | -1.823 | -0.704 | 4.929 | 0.050* | 0.33 | 1.017 | 0.316 | 1.356 | 0.271 | 0.12 |
| Cz | -1.980 | -0.773 | 4.958 | 0.050* | 0.33 | 1.276 | 0.389 | 1.560 | 0.240 | 0.13 |
| C2 | -1.787 | -0.674 | 4.998 | 0.049* | 0.33 | 1.111 | 0.231 | 1.763 | 0.214 | 0.15 |
| C4 | -1.502 | -0.429 | 3.796 | 0.080 | 0.28 | 0.845 | 0.177 | 0.994 | 0.342 | 0.09 |
| CP3 | -1.294 | -0.402 | 3.846 | 0.078 | 0.28 | 0.905 | -0.001 | 3.958 | 0.075 | 0.28 |
| CP1 | -1.480 | -0.399 | 4.089 | 0.071 | 0.29 | 1.114 | 0.096 | 2.724 | 0.130 | 0.21 |
| CP2 | -1.381 | -0.324 | 4.962 | 0.050* | 0.33 | 1.257 | 0.129 | 2.287 | 0.161 | 0.19 |
| CP4 | -1.326 | -0.131 | 4.958 | 0.050* | 0.33 | 0.915 | -0.018 | 1.894 | 0.199 | 0.16 |
| P3 | -0.679 | 0.063 | 2.542 | 0.142 | 0.20 | 0.988 | -0.021 | 4.958 | 0.050* | 0.33 |
| P1 | -0.877 | -0.017 | 2.736 | 0.129 | 0.21 | 1.258 | -0.047 | 5.105 | 0.047* | 0.34 |
| Pz | -1.016 | 0.027 | 3.452 | 0.093 | 0.26 | 1.390 | -0.067 | 4.360 | 0.063 | 0.30 |
| P2 | -1.044 | -0.057 | 2.566 | 0.140 | 0.20 | 1.233 | -0.249 | 4.107 | 0.070 | 0.29 |
| P4 | -1.027 | -0.095 | 2.311 | 0.159 | 0.19 | 0.923 | -0.293 | 2.727 | 0.130 | 0.21 |
| PO3 | -0.493 | -0.134 | 0.564 | 0.470 | 0.05 | 0.767 | -0.371 | 7.296 | 0.022* | 0.42 |
| PO1 | -0.525 | 0.029 | 1.116 | 0.316 | 0.10 | 0.984 | -0.317 | 6.335 | 0.031* | 0.39 |
| POz | -0.579 | 0.173 | 1.873 | 0.201 | 0.16 | 1.135 | -0.470 | 7.607 | 0.020* | 0.43 |
| PO2 | -0.740 | 0.165 | 2.484 | 0.146 | 0.20 | 0.844 | -0.618 | 4.220 | 0.067 | 0.30 |
| PO4 | -0.993 | -0.464 | 0.837 | 0.382 | 0.08 | 0.721 | -0.762 | 4.935 | 0.050* | 0.33 |

**Supplementary Table 1.2.** Experiment 1 (minimal context) – Subsample 2 (N=11): Mean amplitude (µV) for the metaphorical (M) and literal (L) conditions in the N400 and P600 time windows on a sample of relevant electrodes, with significance values for the Metaphoricity factor (F (1,10); * p < 0.05; ** p < 0.01).

| | 320-440 ms | | | | | 550-700 ms | | | | |
|---|---|---|---|---|---|---|---|---|---|---|
| Channel | M (µV) | L (µV) | F value | p | $\eta_p^2$ | M (µV) | L (µV) | F value | p | $\eta_p^2$ |
| F3 | -1.913 | -1.290 | 1.698 | 0.222 | 0.15 | 0.119 | -0.725 | 2.302 | 0.160 | 0.19 |
| Fz | -1.979 | -0.799 | 4.988 | 0.050* | 0.33 | 0.478 | -0.201 | 1.428 | 0.260 | 0.12 |
| F4 | -2.062 | -0.970 | 3.850 | 0.078 | 0.28 | 0.480 | -0.179 | 2.173 | 0.171 | 0.18 |
| FC1 | -2.005 | -0.863 | 5.002 | 0.049* | 0.33 | 0.847 | -0.047 | 1.653 | 0.228 | 0.14 |
| FCz | -2.192 | -0.634 | 6.744 | 0.027* | 0.40 | 0.927 | 0.177 | 1.108 | 0.317 | 0.10 |
| FC2 | -2.169 | -0.667 | 6.687 | 0.027* | 0.40 | 0.928 | 0.279 | 1.140 | 0.311 | 0.10 |
| C3 | -1.810 | -0.711 | 5.000 | 0.049* | 0.33 | 0.460 | -0.152 | 1.391 | 0.266 | 0.12 |
| C1 | -2.000 | -0.818 | 5.362 | 0.043* | 0.35 | 0.876 | 0.050 | 1.636 | 0.230 | 0.14 |
| Cz | -2.171 | -0.865 | 5.122 | 0.047* | 0.34 | 1.186 | 0.191 | 1.619 | 0.232 | 0.14 |
| C2 | -1.960 | -0.770 | 5.009 | 0.049* | 0.33 | 1.170 | 0.135 | 2.368 | 0.155 | 0.19 |
| C4 | -1.701 | -0.519 | 4.935 | 0.051 | 0.33 | 0.997 | 0.138 | 1.828 | 0.206 | 0.15 |
| CP3 | -1.438 | -0.523 | 3.619 | 0.086 | 0.27 | 0.600 | -0.336 | 4.200 | 0.068 | 0.30 |
| CP1 | -1.583 | -0.500 | 3.855 | 0.078 | 0.28 | 0.906 | -0.133 | 2.512 | 0.144 | 0.20 |
| CP2 | -1.448 | -0.378 | 4.966 | 0.050* | 0.33 | 1.227 | 0.025 | 2.409 | 0.152 | 0.19 |
| CP4 | -1.193 | -0.050 | 4.972 | 0.050* | 0.33 | 0.883 | -0.027 | 1.748 | 0.216 | 0.15 |
| P3 | -0.889 | -0.137 | 2.383 | 0.154 | 0.19 | 0.795 | -0.349 | 5.976 | 0.035* | 0.37 |
| P1 | -1.019 | -0.241 | 1.986 | 0.189 | 0.17 | 1.053 | -0.260 | 4.978 | 0.050* | 0.33 |
| Pz | -1.151 | -0.262 | 2.221 | 0.167 | 0.18 | 1.185 | -0.184 | 3.516 | 0.090 | 0.26 |
| P2 | -0.874 | -0.188 | 1.184 | 0.302 | 0.11 | 0.987 | -0.299 | 2.743 | 0.129 | 0.22 |
| P4 | -0.830 | 0.010 | 1.831 | 0.206 | 0.15 | 0.777 | -0.352 | 2.283 | 0.162 | 0.19 |
| PO3 | -0.391 | -0.196 | 0.151 | 0.706 | 0.01 | 0.747 | -0.527 | 7.288 | 0.022* | 0.42 |
| PO1 | -0.719 | -0.235 | 0.828 | 0.384 | 0.08 | 0.903 | -0.393 | 5.502 | 0.041* | 0.35 |
| POz | -0.780 | -0.355 | 0.461 | 0.513 | 0.04 | 0.950 | -0.405 | 4.975 | 0.050* | 0.33 |
| PO2 | -0.910 | -0.263 | 1.066 | 0.326 | 0.10 | 0.690 | -0.460 | 2.449 | 0.149 | 0.20 |
| PO4 | -0.710 | -0.315 | 0.429 | 0.527 | 0.04 | 0.592 | -0.754 | 3.743 | 0.082 | 0.27 |

**Supplementary Table 1.3.** Experiment 2 (supportive context) – Subsample 1 (N=11): Mean amplitude (μV) for the metaphorical (M) and literal (L) conditions in the N400 and P600 time windows on a sample of relevant electrodes, with significance values for the Metaphoricity factor (F (1,10); * p < 0.05; ** p < 0.01).

| | 320-440 ms | | | | | 550-700 ms | | | | |
|---|---|---|---|---|---|---|---|---|---|---|
| Channel | M (μV) | L (μV) | F value | p | $\eta_p^2$ | M (μV) | L (μV) | F value | p | $\eta_p^2$ |
| F3 | -0.694 | -1.252 | 0.913 | 0.362 | 0.08 | 0.243 | -0.949 | 3.270 | 0.101 | 0.25 |
| Fz | -0.593 | -0.715 | 0.035 | 0.855 | 0.00 | 0.483 | -0.980 | 6.614 | 0.028* | 0.40 |
| F4 | -0.938 | -0.510 | 0.442 | 0.521 | 0.04 | 0.120 | -1.063 | 4.229 | 0.067 | 0.30 |
| FC1 | -0.638 | -0.506 | 0.036 | 0.853 | 0.00 | 1.101 | -0.376 | 5.620 | 0.039* | 0.36 |
| FCz | -0.596 | -0.344 | 0.132 | 0.724 | 0.01 | 1.116 | -0.431 | 8.237 | 0.017* | 0.45 |
| FC2 | -0.808 | -0.240 | 0.664 | 0.434 | 0.06 | 0.887 | -0.480 | 7.494 | 0.021* | 0.43 |
| C3 | -0.338 | -0.081 | 0.223 | 0.647 | 0.02 | 1.144 | 0.005 | 4.939 | 0.050* | 0.33 |
| C1 | -0.544 | -0.116 | 0.404 | 0.539 | 0.04 | 1.228 | -0.008 | 5.624 | 0.039* | 0.36 |
| Cz | -0.741 | -0.150 | 0.558 | 0.472 | 0.05 | 1.267 | -0.102 | 7.352 | 0.022* | 0.42 |
| C2 | -0.744 | -0.002 | 1.011 | 0.338 | 0.09 | 1.179 | -0.176 | 6.181 | 0.032* | 0.38 |
| C4 | -0.591 | 0.148 | 1.283 | 0.284 | 0.11 | 1.050 | -0.316 | 6.422 | 0.030* | 0.39 |
| CP3 | 0.102 | 0.419 | 0.365 | 0.559 | 0.04 | 1.211 | 0.067 | 5.268 | 0.045* | 0.35 |
| CP1 | -0.068 | 0.419 | 0.547 | 0.476 | 0.05 | 1.184 | 0.056 | 4.381 | 0.063 | 0.30 |
| CP2 | -0.258 | 0.369 | 0.935 | 0.356 | 0.09 | 1.210 | -0.226 | 8.142 | 0.017* | 0.45 |
| CP4 | -0.249 | 0.312 | 1.105 | 0.318 | 0.10 | 1.239 | -0.500 | 11.351 | 0.007** | 0.53 |
| P3 | 0.476 | 0.843 | 0.720 | 0.416 | 0.07 | 1.233 | -0.034 | 7.765 | 0.019* | 0.44 |
| P1 | 0.410 | 0.863 | 0.761 | 0.404 | 0.07 | 1.210 | 0.059 | 4.564 | 0.058 | 0.31 |
| Pz | 0.259 | 0.995 | 1.743 | 0.216 | 0.15 | 1.194 | -0.034 | 5.465 | 0.042* | 0.35 |
| P2 | 0.227 | 0.796 | 1.218 | 0.296 | 0.11 | 1.192 | -0.321 | 6.107 | 0.033* | 0.38 |
| P4 | -0.006 | 0.614 | 1.292 | 0.282 | 0.11 | 1.130 | -0.494 | 7.102 | 0.024* | 0.42 |
| PO3 | 0.186 | 0.804 | 2.866 | 0.121 | 0.22 | 0.952 | 0.097 | 2.240 | 0.165 | 0.18 |
| PO1 | 0.247 | 0.940 | 2.716 | 0.130 | 0.21 | 0.943 | -0.187 | 2.495 | 0.145 | 0.20 |
| POz | 0.450 | 1.205 | 2.899 | 0.119 | 0.22 | 1.218 | -0.058 | 4.162 | 0.069 | 0.29 |
| PO2 | 0.323 | 1.030 | 2.202 | 0.169 | 0.18 | 1.021 | -0.401 | 4.139 | 0.069 | 0.29 |
| PO4 | -0.256 | 0.446 | 2.612 | 0.137 | 0.21 | 0.808 | -0.544 | 3.464 | 0.092 | 0.26 |

**Supplementary Table 1.4.** Experiment 2 (supportive context) – Subsample 2 (N=11): Mean amplitude (µV) for the metaphorical (M) and literal (L) conditions in the N400 and P600 time windows on a sample of relevant electrodes, with significance values for the Metaphoricity factor (F (1,10); * p < 0.05; ** p < 0.01).

| | 320-440 ms | | | | | 550-700 ms | | | | |
|---|---|---|---|---|---|---|---|---|---|---|
| Channel | M (µV) | L (µV) | F value | p | $\eta_p^2$ | M (µV) | L (µV) | F value | p | $\eta_p^2$ |
| F3 | -1.121 | -1.305 | 0.121 | 0.736 | 0.01 | -0.034 | -1.147 | 3.001 | 0.114 | 0.23 |
| Fz | -0.936 | -0.613 | 0.418 | 0.532 | 0.04 | 0.349 | -0.986 | 6.347 | 0.030* | 0.39 |
| F4 | -1.185 | -0.439 | 2.113 | 0.177 | 0.17 | 0.050 | -0.915 | 4.949 | 0.050* | 0.33 |
| FC1 | -0.990 | -0.448 | 0.974 | 0.347 | 0.09 | 0.856 | -0.378 | 4.962 | 0.050* | 0.33 |
| FCz | -0.909 | -0.212 | 1.814 | 0.208 | 0.15 | 0.915 | -0.358 | 7.712 | 0.020* | 0.44 |
| FC2 | -1.125 | -0.114 | 3.957 | 0.075 | 0.28 | 0.814 | -0.300 | 6.949 | 0.025* | 0.41 |
| C3 | -0.550 | -0.070 | 1.283 | 0.284 | 0.11 | 0.765 | -0.174 | 3.264 | 0.101 | 0.25 |
| C1 | -0.858 | -0.052 | 2.802 | 0.125 | 0.22 | 1.032 | 0.001 | 5.008 | 0.049* | 0.33 |
| Cz | -1.062 | -0.015 | 3.687 | 0.084 | 0.27 | 1.234 | 0.074 | 5.918 | 0.035* | 0.37 |
| C2 | -0.952 | 0.170 | 4.812 | 0.053 | 0.32 | 1.099 | 0.136 | 5.002 | 0.049* | 0.33 |
| C4 | -0.820 | 0.222 | 4.613 | 0.057 | 0.32 | 0.935 | 0.029 | 4.998 | 0.049* | 0.33 |
| CP3 | -0.086 | 0.332 | 1.097 | 0.320 | 0.10 | 0.925 | 0.044 | 3.133 | 0.107 | 0.24 |
| CP1 | -0.305 | 0.518 | 3.188 | 0.104 | 0.24 | 1.059 | 0.145 | 2.888 | 0.120 | 0.22 |
| CP2 | -0.449 | 0.542 | 5.962 | 0.035* | 0.37 | 1.215 | 0.124 | 5.051 | 0.048* | 0.34 |
| CP4 | -0.201 | 0.594 | 3.872 | 0.077 | 0.28 | 1.161 | -0.123 | 6.569 | 0.028* | 0.40 |
| P3 | 0.243 | 0.718 | 2.291 | 0.161 | 0.19 | 1.038 | 0.011 | 4.958 | 0.050* | 0.33 |
| P1 | 0.207 | 0.850 | 2.913 | 0.119 | 0.23 | 1.085 | 0.224 | 2.740 | 0.129 | 0.22 |
| Pz | 0.093 | 1.012 | 5.672 | 0.039* | 0.36 | 1.226 | 0.265 | 3.238 | 0.102 | 0.24 |
| P2 | 0.167 | 0.834 | 2.972 | 0.115 | 0.23 | 1.149 | 0.108 | 4.956 | 0.050* | 0.33 |
| P4 | 0.086 | 0.829 | 3.186 | 0.105 | 0.24 | 1.069 | -0.061 | 5.124 | 0.047* | 0.34 |
| PO3 | 0.340 | 0.923 | 3.487 | 0.091 | 0.26 | 0.855 | 0.265 | 1.118 | 0.315 | 0.10 |
| PO1 | 0.084 | 0.591 | 1.247 | 0.290 | 0.11 | 0.982 | 0.377 | 1.054 | 0.329 | 0.10 |
| POz | 0.322 | 1.112 | 4.862 | 0.052 | 0.33 | 1.243 | 0.301 | 2.023 | 0.185 | 0.17 |
| PO2 | 0.206 | 0.849 | 2.662 | 0.134 | 0.21 | 1.127 | 0.151 | 1.888 | 0.199 | 0.16 |
| PO4 | 0.077 | 0.660 | 2.046 | 0.183 | 0.17 | 0.843 | 0.024 | 1.289 | 0.283 | 0.11 |

# Supplementary Tables 2. Analysis with familiarity

**Supplementary Table 2.1.** Experiment 1 (minimal context): Mean amplitude (µV) for the familiar metaphor (Mfam), non-familiar metaphor (Mnfam) and literal (L) conditions in the N400 and P600 time windows on a sample of relevant electrodes, with significance values for the Metaphoricity factor (F (2,24); * p < 0.05; ** p < 0.01).

| | 320-440 ms | | | | | | 550-700 ms | | | | | |
|---|---|---|---|---|---|---|---|---|---|---|---|---|
| Channel | Mfam (µV) | Mnfam (µV) | L (µV) | F value | p | $\eta_p^2$ | Mfam (µV) | Mnfam (µV) | L (µV) | F value | p | $\eta_p^2$ |
| F3 | -2.087 | -1.655 | -1.159 | 2.042 | 0.152 | 0.15 | 0.234 | -0.219 | -0.491 | 0.598 | 0.558 | 0.05 |
| Fz | -2.363 | -1.437 | -0.719 | 4.821 | 0.017* | 0.29 | 0.383 | 0.096 | -0.113 | 0.257 | 0.775 | 0.02 |
| F4 | -2.690 | -1.237 | -0.811 | 6.192 | 0.007** | 0.34 | 0.174 | 0.153 | -0.180 | 0.205 | 0.737 | 0.02 |
| FC1 | -2.176 | -1.807 | -0.904 | 3.469 | 0.047* | 0.22 | 0.879 | 0.727 | 0.074 | 0.683 | 0.515 | 0.05 |
| FCz | -2.434 | -1.805 | -0.714 | 5.051 | 0.015* | 0.30 | 0.860 | 0.751 | 0.278 | 0.326 | 0.725 | 0.03 |
| FC2 | -2.624 | -1.606 | -0.691 | 6.544 | 0.005** | 0.35 | 0.693 | 0.719 | 0.303 | 0.209 | 0.729 | 0.02 |
| C3 | -1.833 | -1.785 | -0.795 | 2.913 | 0.074 | 0.20 | 0.664 | 0.381 | 0.108 | 0.400 | 0.675 | 0.03 |
| C1 | -2.147 | -1.894 | -0.917 | 4.217 | 0.027* | 0.36 | 1.070 | 0.758 | 0.170 | 0.923 | 0.411 | 0.07 |
| Cz | -2.490 | -1.879 | -0.985 | 3.985 | 0.032* | 0.35 | 1.318 | 1.040 | 0.280 | 1.012 | 0.378 | 0.08 |
| C2 | -2.449 | -1.490 | -0.837 | 5.159 | 0.014* | 0.30 | 1.113 | 0.959 | 0.189 | 0.903 | 0.419 | 0.07 |
| C4 | -2.310 | -1.053 | -0.564 | 6.532 | 0.005** | 0.35 | 0.806 | 0.762 | 0.139 | 0.540 | 0.590 | 0.04 |
| CP3 | -1.336 | -1.478 | -0.533 | 2.787 | 0.082 | 0.19 | 0.787 | 0.592 | -0.153 | 1.564 | 0.230 | 0.12 |
| CP1 | -1.656 | -1.550 | -0.603 | 2.860 | 0.077 | 0.19 | 1.110 | 0.824 | -0.036 | 1.481 | 0.247 | 0.11 |
| CP2 | -1.763 | -1.206 | -0.483 | 3.684 | 0.040* | 0.23 | 1.276 | 1.030 | 0.076 | 1.368 | 0.274 | 0.10 |
| CP4 | -1.743 | -1.048 | -0.261 | 4.230 | 0.027* | 0.36 | 0.766 | 0.721 | -0.065 | 0.874 | 0.430 | 0.07 |
| P3 | -0.571 | -0.944 | -0.074 | 1.867 | 0.176 | 0.13 | 1.014 | 0.734 | -0.175 | 2.964 | 0.071 | 0.20 |
| P1 | -0.832 | -1.125 | -0.245 | 1.679 | 0.208 | 0.12 | 1.434 | 0.873 | -0.140 | 3.231 | 0.057 | 0.21 |
| Pz | -1.241 | -1.111 | -0.281 | 2.133 | 0.140 | 0.15 | 1.457 | 1.025 | -0.115 | 2.469 | 0.106 | 0.17 |
| P2 | -1.213 | -0.927 | -0.302 | 1.469 | 0.250 | 0.11 | 1.254 | 0.778 | -0.294 | 2.300 | 0.122 | 0.16 |
| P4 | -1.401 | -0.792 | -0.228 | 2.441 | 0.108 | 0.17 | 0.809 | 0.541 | -0.393 | 1.472 | 0.250 | 0.11 |
| PO3 | 0.050 | -0.715 | -0.114 | 1.134 | 0.338 | 0.09 | 1.072 | 0.517 | -0.415 | 5.246 | 0.013* | 0.30 |
| PO1 | -0.284 | -0.995 | -0.162 | 1.370 | 0.273 | 0.10 | 1.323 | 0.562 | -0.368 | 4.630 | 0.020* | 0.28 |
| POz | -0.559 | -1.005 | -0.305 | 0.744 | 0.486 | 0.06 | 1.420 | 0.649 | -0.435 | 4.278 | 0.026* | 0.26 |
| PO2 | -0.844 | -1.047 | -0.265 | 0.930 | 0.408 | 0.07 | 1.168 | 0.313 | -0.572 | 2.969 | 0.070 | 0.20 |
| PO4 | -1.119 | -1.106 | -0.663 | 0.415 | 0.665 | 0.03 | 0.752 | 0.284 | -0.839 | 2.752 | 0.084 | 0.19 |

**Supplementary Table 2.2.** Experiment 1 (minimal context): Planned contrasts between familiar metaphors (Mfam) and non-familiar metaphors (Mnfam), and between the two metaphorical conditions together (Mfam + Mnfam) and the literal (L) condition in the N400 and P600 time windows on a sample of relevant electrodes, with significance values (F (1,12); * p < 0.05; ** p < 0.01). Light blue background indicates channels where the effect was significant in Table 2.1.

| | 320-440 ms | | | | 550-700 ms | | | |
|---|---|---|---|---|---|---|---|---|
| | Mfam vs Mnfam | | (Mfam + Mnfam) vs L | | Mfam vs Mnfam | | (Mfam + Mnfam) vs L | |
| Channel | F value | p | F value | p | F value | p | F value | p |
| F3 | 0.908 | 0.359 | 3.114 | 0.103 | 0.433 | 0.523 | 0.782 | 0.394 |
| Fz | 3.103 | 0.104 | 6.479 | 0.026* | 0.148 | 0.707 | 0.406 | 0.536 |
| F4 | 6.428 | 0.026* | 5.932 | 0.031* | 0.001 | 0.977 | 0.597 | 0.455 |
| FC1 | 0.713 | 0.415 | 5.199 | 0.042* | 0.039 | 0.846 | 1.473 | 0.248 |
| FCz | 1.637 | 0.225 | 7.346 | 0.019* | 0.018 | 0.895 | 0.709 | 0.416 |
| FC2 | 4.245 | 0.062 | 8.258 | 0.014* | 0.001 | 0.975 | 0.542 | 0.476 |
| C3 | 0.010 | 0.923 | 5.890 | 0.032* | 0.173 | 0.685 | 0.738 | 0.407 |
| C1 | 0.434 | 0.522 | 6.433 | 0.026* | 0.195 | 0.667 | 1.822 | 0.202 |
| Cz | 1.568 | 0.234 | 5.690 | 0.034* | 0.137 | 0.718 | 1.871 | 0.196 |
| C2 | 3.986 | 0.069 | 6.128 | 0.029* | 0.037 | 0.850 | 2.126 | 0.171 |
| C4 | 7.530 | 0.018* | 5.801 | 0.033* | 0.003 | 0.955 | 1.312 | 0.274 |
| CP3 | 0.129 | 0.726 | 4.704 | 0.051 | 0.091 | 0.768 | 4.427 | 0.057 |
| CP1 | 0.063 | 0.805 | 4.544 | 0.054 | 0.148 | 0.707 | 3.261 | 0.096 |
| CP2 | 1.809 | 0.204 | 4.854 | 0.048* | 0.101 | 0.756 | 2.687 | 0.127 |
| CP4 | 2.383 | 0.149 | 5.410 | 0.038* | 0.004 | 0.952 | 1.883 | 0.195 |
| P3 | 0.762 | 0.400 | 2.763 | 0.122 | 0.259 | 0.620 | 6.710 | 0.024* |
| P1 | 0.459 | 0.511 | 2.467 | 0.142 | 0.715 | 0.414 | 6.411 | 0.026* |
| Pz | 0.101 | 0.756 | 3.141 | 0.102 | 0.332 | 0.575 | 4.845 | 0.048* |
| P2 | 0.407 | 0.535 | 2.015 | 0.181 | 0.433 | 0.523 | 4.015 | 0.068 |
| P4 | 1.862 | 0.197 | 2.757 | 0.123 | 0.135 | 0.720 | 2.768 | 0.122 |
| PO3 | 1.763 | 0.209 | 0.264 | 0.617 | 1.413 | 0.258 | 9.183 | 0.010* |
| PO1 | 1.567 | 0.234 | 1.133 | 0.308 | 1.761 | 0.209 | 7.879 | 0.016* |
| POz | 0.664 | 0.431 | 0.808 | 0.386 | 1.335 | 0.270 | 7.864 | 0.016* |
| PO2 | 0.125 | 0.730 | 1.640 | 0.224 | 1.529 | 0.240 | 4.240 | 0.062 |
| PO4 | 0.001 | 0.981 | 0.779 | 0.395 | 0.453 | 0.514 | 5.020 | 0.045* |

**Supplementary Table 2.3.** Experiment 2 (supportive context): Mean amplitude (µV) for the familiar metaphor (Mfam), non-familiar metaphor (Mnfam) and literal (L) conditions in the N400 and P600 time windows on a sample of relevant electrodes, with significance values for the Metaphoricity factor (F (2,24); * p < 0.05; ** p < 0.01).

| | 320-440 ms | | | | | | 550-700 ms | | | | | |
|---|---|---|---|---|---|---|---|---|---|---|---|---|
| Channel | Mfam (µV) | Mnfam (µV) | L (µV) | F value | p | $\eta_p^2$ | Mfam (µV) | Mnfam (µV) | L (µV) | F value | p | $\eta_p^2$ |
| F3 | -1.187 | -0.822 | -1.475 | 0.855 | 0.438 | 0.07 | -0.025 | 0.725 | -1.031 | 4.281 | 0.026* | 0.26 |
| Fz | -0.838 | -0.774 | -0.907 | 0.026 | 0.975 | 0.00 | 0.305 | 0.968 | -1.025 | 5.419 | 0.011* | 0.31 |
| F4 | -0.959 | -1.150 | -0.664 | 0.270 | 0.766 | 0.02 | 0.004 | 0.564 | -1.026 | 3.109 | 0.063 | 0.21 |
| FC1 | -0.778 | -1.002 | -0.770 | 0.093 | 0.911 | 0.01 | 1.193 | 1.135 | -0.422 | 4.049 | 0.031* | 0.25 |
| FCz | -0.748 | -0.915 | -0.589 | 0.118 | 0.889 | 0.01 | 1.183 | 1.202 | -0.461 | 3.950 | 0.033* | 0.25 |
| FC2 | -0.805 | -1.206 | -0.462 | 0.588 | 0.563 | 0.05 | 1.012 | 1.019 | -0.472 | 3.439 | 0.049* | 0.22 |
| C3 | -0.257 | -0.868 | -0.336 | 0.737 | 0.489 | 0.06 | 1.028 | 1.102 | -0.095 | 3.130 | 0.062 | 0.21 |
| C1 | -0.539 | -1.066 | -0.387 | 0.567 | 0.575 | 0.05 | 1.361 | 1.102 | -0.069 | 3.198 | 0.059 | 0.21 |
| Cz | -0.625 | -1.351 | -0.408 | 0.789 | 0.466 | 0.06 | 1.694 | 1.044 | -0.084 | 3.408 | 0.050* | 0.22 |
| C2 | -0.462 | -1.303 | -0.189 | 1.092 | 0.352 | 0.08 | 1.479 | 1.009 | -0.124 | 2.673 | 0.090 | 0.18 |
| C4 | -0.291 | -1.176 | -0.047 | 1.435 | 0.258 | 0.11 | 1.116 | 0.874 | -0.298 | 2.499 | 0.103 | 0.17 |
| CP3 | 0.390 | -0.511 | 0.167 | 1.089 | 0.352 | 0.08 | 1.113 | 1.074 | 0.004 | 2.549 | 0.099 | 0.18 |
| CP1 | 0.163 | -0.733 | 0.183 | 0.988 | 0.387 | 0.08 | 1.288 | 1.029 | 0.016 | 2.370 | 0.115 | 0.16 |
| CP2 | 0.194 | -1.095 | 0.174 | 1.759 | 0.194 | 0.13 | 1.584 | 0.748 | -0.186 | 2.935 | 0.072 | 0.20 |
| CP4 | 0.343 | -1.062 | 0.190 | 2.513 | 0.102 | 0.17 | 1.490 | 0.617 | -0.490 | 4.033 | 0.031* | 0.25 |
| P3 | 0.973 | -0.254 | 0.668 | 2.459 | 0.107 | 0.17 | 1.329 | 0.883 | -0.048 | 3.073 | 0.065 | 0.20 |
| P1 | 0.818 | -0.409 | 0.622 | 1.862 | 0.177 | 0.13 | 1.375 | 0.845 | 0.039 | 2.039 | 0.152 | 0.15 |
| Pz | 0.658 | -0.638 | 0.677 | 2.054 | 0.150 | 0.15 | 1.628 | 0.692 | -0.025 | 2.276 | 0.124 | 0.16 |
| P2 | 0.696 | -0.681 | 0.482 | 2.018 | 0.155 | 0.14 | 1.612 | 0.511 | -0.293 | 2.883 | 0.075 | 0.19 |
| P4 | 0.704 | -0.947 | 0.445 | 3.623 | 0.042* | 0.23 | 1.393 | 0.401 | -0.514 | 3.551 | 0.045* | 0.23 |
| PO3 | 1.211 | -0.428 | 0.857 | 4.728 | 0.036* | 0.28 | 1.303 | 0.497 | 0.141 | 2.146 | 0.139 | 0.15 |
| PO1 | 0.821 | -0.542 | 0.533 | 2.509 | 0.102 | 0.17 | 1.012 | 0.759 | -0.100 | 1.419 | 0.262 | 0.11 |
| POz | 0.917 | -0.514 | 0.875 | 2.985 | 0.070 | 0.20 | 1.418 | 0.745 | -0.011 | 1.584 | 0.226 | 0.12 |
| PO2 | 0.855 | -0.756 | 0.643 | 3.411 | 0.050* | 0.22 | 1.359 | 0.436 | -0.298 | 2.090 | 0.146 | 0.15 |
| PO4 | 0.660 | -1.397 | 0.192 | 6.309 | 0.006** | 0.34 | 1.152 | -0.035 | -0.515 | 2.586 | 0.096 | 0.18 |

**Supplementary Table 2.4.** Experiment 2 (supportive context): Planned contrasts between familiar metaphors (Mfam) and non-familiar metaphors (Mnfam), and between the two metaphorical conditions together (Mfam + Mnfam) and the literal (L) condition in the N400 and P600 time windows on a sample of relevant electrodes, with significance values (F (1,12); * p < 0.05; ** p < 0.01). Light blue background indicates channels where the effect was significant in Table 2.3.

| | 320-440 ms | | | | 550-700 ms | | | |
|---|---|---|---|---|---|---|---|---|
| | Mfam vs Mnfam | | (Mfam + Mnfam) vs L | | Mfam vs Mnfam | | (Mfam + Mnfam) vs L | |
| Channel | F value | p | F value | p | F value | p | F value | p |
| F3 | 0.785 | 0.393 | 0.891 | 0.364 | 1.913 | 0.192 | 5.894 | 0.032* |
| Fz | 0.013 | 0.910 | 0.035 | 0.854 | 1.015 | 0.334 | 11.230 | 0.006** |
| F4 | 0.071 | 0.795 | 0.543 | 0.475 | 0.614 | 0.448 | 7.035 | 0.021* |
| FC1 | 0.165 | 0.692 | 0.043 | 0.838 | 0.007 | 0.933 | 8.980 | 0.011* |
| FCz | 0.060 | 0.811 | 0.180 | 0.679 | 0.001 | 0.981 | 12.930 | 0.004** |
| FC2 | 0.313 | 0.586 | 0.914 | 0.358 | 0.000 | 0.993 | 11.622 | 0.005** |
| C3 | 1.128 | 0.309 | 0.254 | 0.623 | 0.019 | 0.893 | 6.324 | 0.027* |
| C1 | 0.566 | 0.466 | 0.568 | 0.465 | 0.151 | 0.704 | 7.999 | 0.015* |
| Cz | 0.772 | 0.397 | 0.811 | 0.386 | 0.627 | 0.444 | 10.185 | 0.008** |
| C2 | 0.939 | 0.352 | 1.332 | 0.271 | 0.318 | 0.583 | 7.795 | 0.016* |
| C4 | 1.302 | 0.276 | 1.646 | 0.224 | 0.098 | 0.760 | 7.078 | 0.021* |
| CP3 | 1.467 | 0.249 | 0.271 | 0.612 | 0.004 | 0.949 | 5.766 | 0.033* |
| CP1 | 1.115 | 0.312 | 0.753 | 0.403 | 0.153 | 0.703 | 5.355 | 0.039* |
| CP2 | 1.900 | 0.193 | 1.420 | 0.256 | 0.904 | 0.361 | 8.262 | 0.014* |
| CP4 | 2.837 | 0.118 | 1.611 | 0.228 | 1.140 | 0.307 | 10.311 | 0.007** |
| P3 | 3.060 | 0.106 | 0.738 | 0.407 | 0.503 | 0.492 | 7.161 | 0.020* |
| P1 | 2.202 | 0.164 | 0.930 | 0.354 | 0.508 | 0.490 | 4.584 | 0.053 |
| Pz | 2.034 | 0.179 | 2.113 | 0.172 | 1.003 | 0.336 | 5.585 | 0.036* |
| P2 | 2.245 | 0.160 | 1.232 | 0.289 | 1.454 | 0.251 | 5.623 | 0.035* |
| P4 | 4.515 | 0.055 | 1.617 | 0.228 | 1.671 | 0.220 | 6.097 | 0.030* |
| PO3 | 5.398 | 0.039* | 2.192 | 0.164 | 2.081 | 0.175 | 2.205 | 0.163 |
| PO1 | 3.249 | 0.097 | 0.824 | 0.382 | 0.158 | 0.698 | 2.344 | 0.152 |
| POz | 2.889 | 0.115 | 3.366 | 0.091 | 0.542 | 0.476 | 3.488 | 0.086 |
| PO2 | 3.779 | 0.076 | 2.219 | 0.162 | 1.126 | 0.309 | 3.386 | 0.091 |
| PO4 | 7.578 | 0.018* | 2.345 | 0.152 | 2.525 | 0.138 | 2.646 | 0.130 |
